# New target prediction and visualization tools incorporating open source molecular fingerprints for TB Mobile 2.0

**DOI:** 10.1186/s13321-014-0038-2

**Published:** 2014-08-04

**Authors:** Alex M Clark, Malabika Sarker, Sean Ekins

**Affiliations:** 1 Molecular Materials Informatics, 1900 St. Jacques #302, Montreal H3J 2S1, Quebec, Canada; 2 SRI International, 333 Ravenswood Avenue, Menlo Park 94025, CA, USA; 3 Collaborative Drug Discovery, 1633 Bayshore Highway, Suite 342, Burlingame 94010, CA, USA; 4 Collaborations in Chemistry, 5616 Hilltop Needmore Road, Fuquay-Varina 27526, NC, USA

**Keywords:** Mobile app, Mycobacterium tuberculosis, TB mobile, Tuberculosis, Target prediction

## Abstract

**Background:**

We recently developed a freely available mobile app (*TB Mobile*) for both iOS and Android platforms that displays *Mycobacterium tuberculosis* (*Mtb*) active molecule structures and their targets with links to associated data. The app was developed to make target information available to as large an audience as possible.

**Results:**

We now report a major update of the iOS version of the app. This includes enhancements that use an implementation of ECFP_6 fingerprints that we have made open source. Using these fingerprints, the user can propose compounds with possible anti-TB activity, and view the compounds within a cluster landscape. Proposed compounds can also be compared to existing target data, using a näive Bayesian scoring system to rank probable targets. We have curated an additional 60 new compounds and their targets for *Mtb* and added these to the original set of 745 compounds. We have also curated 20 further compounds (many without targets in TB Mobile) to evaluate this version of the app with 805 compounds and associated targets.

**Conclusions:**

*TB Mobile* can now manage a small collection of compounds that can be imported from external sources, or exported by various means such as email or app-to-app inter-process communication. This means that *TB Mobile* can be used as a node within a growing ecosystem of mobile apps for cheminformatics. It can also cluster compounds and use internal algorithms to help identify potential targets based on molecular similarity. *TB Mobile* represents a valuable dataset, data-visualization aid and target prediction tool.

## Background

Efforts to make data accessible and useful for drug discovery are needed perhaps now more so than ever before. Over the past decade we have seen considerable investments in high throughput screening which adds to the quantity of data available [1]. In particular the focus of our work is on tuberculosis (TB) caused by *Mycobacterium tuberculosis* (*Mtb*). TB infects nearly 33% of the entire world population and causes approximately 1.3 million deaths each year based on the 2013 WHO global tuberculosis report [2]-[5]. Increased incidence of TB in both developing and industrialized countries, including the USA is of concern and exacerbated by the widespread emergence of drug-resistant (multidrug-resistant TB (MDR-TB)) strains [6] and co-infection with the human immunodeficiency virus (HIV). Even more troubling is the emergence of extensively drug-resistant (XDR) TB which is present in nearly 60 countries [7]. The pipeline for TB therapeutics is limited, [8],[9], having produced the first drug in 40 years in 2012 in the form of bedaquiline for multidrug resistant TB [10],[11]. Part of the difficulty in drug discovery has been due to poor success of target-based high-throughput screening [12]. In the last 10 years there has been a marked shift in favor of high-throughput screening in whole cells [13]-[18]. Unfortunately the hit rates of this approach are usually low single-digit (or less) [1],[16],[19],[20] which makes this a very costly and wasteful exercise. In addition, finding hits active in whole cells provides no information on the likely target, which is important to enable drug optimization. Target identification in turn is a generally very slow and a further costly process.

Various efforts have been described for predicting and prioritizing which *Mtb* targets to consider for drug design that represent sophisticated workflows combining methods such as pathway/network analysis, flux balance studies and comparative genomics, structure assessment and binding pocket analysis [21]-[24]. The addition of binding site similarity and docking have also been used to propose targets in the TB proteome for FDA approved drugs [25],[26]. Following whole cell screening, whole-genome sequencing of resistant mutants and recombineering it is possible to identify targets for compounds experimentally [27]. In contrast to these approaches, computational prediction of compounds and their targets (target deconvolution) has involved ligand similarity using Bayesian methods as a domain fishing model [28] and other methods [29]. We previously described [30] how we initially curated >700 molecules with *Mtb* target/s along with various links to the target, genes (tbdb.org), pathways, human homolog information [31] and essentiality data [32]. This information initially comprised a dataset publically available in the Collaborative Drug Discovery (CDD) database [31]. We then used this dataset as the basis to generate a simple mobile app called *TB Mobile*, which is useful for viewing and manipulating data about compounds with activity against *Mtb*, their targets and other related information [30]. We had also used the app to make predictions previously [33],[34] including for a set of open access compounds from GSK [17]. This work preceded that of another group that used chemogenomics space search, structural space search and historical assay space search predict the same compounds [35]. The historical assay space search used was proprietary to GSK so this reported approach and data is not available to other researchers. Also it would appear this group did not take advantage of known ligands and their targets in *Mtb*.

Our previous work on *TB Mobile* demonstrated how the app can be a useful resource to filter by target, essentiality, human homolog and similarity search [30]. The app also retrieves first line drugs that are present in the database as we have previously shown. In addition we generated predictions for an additional 20 compounds for which targets were either known or unknown [30]. This testing pointed out limitations and suggested future versions may use predictive machine learning models [36],[37] for suggesting targets and it would likely require a larger set of molecules to build further confidence. We proposed addition of molecules for targets not currently represented or under-represented would be important as well as balancing the bias towards over-represented targets. At that time we had representatives of 68 targets in *TB Mobile* which is clearly a small fraction of the over 1400 possible targets in *Mtb*[38], but in reality it probably covers the majority of characterized targets adequately and is to our knowledge the most extensive *Mtb* specific database related to small molecules and their targets. However it is important to remember the targets of replicating cells do not overlap with targets of nonreplicating bacteria and the number of candidate target proteins may actually be higher [39]. We now describe our efforts to curate new data, provide new functionality and test the app that is now available as *TB Mobile* version 2.0 for iOS (iPhone, iPod, iPad) [40].

## Methods

### Dataset curation

The process of dataset curation was previously described by us and for updating purposes we performed searches for recent papers describing molecules and known targets in *Mtb.* We manually curated molecules and data combined with URL links to literature and TBDB [41],[42] and these were deposited in the CDD database [31].

### TB mobile app software development: open source fingerprint implementation

A number of modelling projects in recent years have successfully made use of the extended connectivity fingerprints, commonly referred to as ECFP_*n* or FCFP_*n* (*n* = 2, 4 or 6). For example we have experience in apply the FCFP_6 descriptors to modeling phenotypic HTS data for *Mtb*[32],[36],[37],[43]-[49]. These fingerprints are created by enumerating a collection of substructures using breadth-first expansion from a starting atom. The fingerprint method was originally made available as part of the Pipeline Pilot project [50],[51] and similar methods have been made available from ChemAxon’s proprietary JChem [52] and RDKit [53]. The Accelrys fingerprint methodology was published in detail [54], but the disclosure omitted a number of trade secrets, which means that while it is now straightforward to implement an algorithm that generates fingerprints that are similarly effective, it is not possible to produce results that can be directly comparable between the two different implementations.

We have need of a drop-in replacement for the ECFP_6 fingerprints that can be readily ported between multiple toolkits and programming languages. We have therefore built and validated an algorithm that follows the published reference for ECFP and FCFP fingerprints as closely as possible, and made the resulting code available to the public as a feature in the Chemical Development Kit (CDK) project [55],[56], under an open source license. While this is in itself a valuable addition to the popular Java-based toolkit, we have taken care to implement the algorithm in a concise manner with few external dependencies. By avoiding toolkit-specific supporting algorithms, this has allowed us to port the ECFP_6 algorithm to other platforms, in particular the Objective-C programming language used for native iOS apps, with literally comparable results, which is a key prerequisite for the new functionality that is available in the *TB Mobile* app.

In the following section, we describe the ECFP_6 implementation in sufficient detail such that a skilled programmer can precisely re-implement the algorithm. The freely available open source implementation that is part of the CDK can be consulted for guidance, and used to generate validation examples, to ensure that the results are identical. The reference [54] should first be consulted for an instructive overview of the methodology.

### Molecule preparation

The input is assumed to be a molecule that is represented as a connection table with the lowest common denominator feature set of an MDL Molfile, i.e. all atoms are represented as symbols from the periodic table, and all bonds have an integral bond order. Most organic molecules can be represented using bond orders of 1, 2 and 3, but organometallic compounds can also be described with bond orders of 0 or 4 or higher [57]. Aromatic rings must be initially represented in Kekulé form, i.e. alternating single/double bonds.

Implicit hydrogens are optional: the molecule can have some, all or none of its hydrogen atoms listed as individual atoms, as long as the implicit hydrogen counting method described in the algorithm obtains the expected answer. The same identical fingerprints will be created regardless of whether hydrogens are implicit or explicit.

Implicit hydrogens are calculated using a conservative formula: only elements C, N, O, P and S are eligible for auto-calculated implicit hydrogens. The respective formula for each of these cases is:

 carbon **H** = 4 - |**C**| - **U** - **B**

 nitrogen and phosphorus: **H** = 3 - **C** - **U** - **B**

 oxygen and sulfur: **H** = 2 - **C** - **U** - **B**

where **C** is the formal charge, **U** is the number of unpaired electrons standing in lieu of bonds, and **B** is the sum of the bond orders of neighbouring atoms. Note that for carbon, the absolute value of the charge is used. The unpaired electron count is typically listed as 1 for radicals and 2 for carbenes (it does *not* include conventional lone pairs, such as those on oxygen or nitrogen).

Any molecules for which the conservative implicit hydrogen calculation formula does not provide the correct structure must either be submitted using a file format that allows the specification of the number of virtual hydrogen atoms, or the hydrogen atoms for this atom must be created explicitly as individual atom nodes in order that they be counted.

Aromaticity is calculated by enumerating rings of size 6, and labelling only those that are capable of adopting a 6π aromatic alternating double bond forms. This does not include lone-pair aromatics (e.g. thiophene), large rings (e.g. porphyrins), charged 5-rings (e.g. imidazolium) or exocyclics (e.g. cyclic amides). Ring systems such as naphthalene or anthracene are considered aromatic for all rings, since all rings have a qualifying resonance form.

The algorithm proceeds as:

 enumerate all 6-membered rings (excluding those made up of smaller rings)

 evaluate each ring to see if it has alternating single-double bonds; for each qualifying ring, mark the atoms and bonds are aromatic and remove the ring from the list

 loop:

 evaluate each ring for alternating single-double bonds, whereby any bond previously marked as aromatic is considered as a wildcard, i.e. it may be considered as either single or double

 repeat until no more aromatic rings can be found

Stereochemistry may be encoded in a variety of ways, e.g. wedge bonds, chiral parity, CIP labels, as long as the algorithm is capable of making use of this description to derive the correct tetrahedral conformation. 3 otherwise identical molecules with different chiral states (i.e. R, S or unspecified) can usually be expected to deliver 3 different sets of fingerprints, though they will be similar (note however that in some cases the limited reach of the circular fingerprints means that chirality does not resolve to different atom identities, especially for FCFP class fingerprints).

The internal representation used by this algorithm is done by considering every *p*-block element with 4 substituents (one of which may be an *implicit* hydrogen): if it is marked with a chiral parity flag, or has at least one wedge bond, then an attempt will be made to map the 4 substituents onto a tetrahedron:

If the 4 substituents are mapped onto a tetrahedron in an order that is equivalent to the above, the atom indices can be entered into an array in a specific order, e.g. [A, B, C, D] where the letters correspond to an atom index, or *none* in the case of an implicit hydrogen placeholder. For this array of size 4, there are a total of 24 permutations. Each permutation has an *odd* or *even* parity relative to the starting geometry, e.g. [B, A, C, D] is an odd permutation (one swap) while [B, A, D, C] is an even permutation (two swaps). Even permutations refer to an equivalent geometry, i.e. the tetrahedron can be rotated around to its original position, while odd permutations are inequivalent, i.e. if the substituents are different, then it cannot be restored to its original state by rotation, which means it is enantiomeric. Therefore by creating an array [A, B, C, D] that is representative of the geometry, or *any even permutation* of it, the necessary chirality information is encoded. This will be used during the fingerprint generation process. This array is referred to as the *tetrahedral rubric* for the atom. Note that it is valid to generate this for an atom even if it is not actually chiral: the symmetry, or lack thereof, will be resolved during the assignment process.

### ECFP atom identity

For the ECFP-class of fingerprints, each *non-hydrogen* atom is assigned an initial identity which is made up by first determining the following properties:

**N** = number of heavy atom neighbours

**H** = number of hydrogen neighbours (implicit and explicit)

**D** = atom valence - number of hydrogens (implicit and explicit)

**A** = atomic number

**C** = formal ionic charge (integer)

**R** = 1 if the atom is in a ring (of any size), 0 otherwise

The initial hash code of each atom is composed by adding the following bytes to a CRC32 calculation:

 (**N** < < 4) | **D**

A

**C** + 0x80

 (**H** < < 4) | **R**

The CRC32 calculation is the same method used by PNG and ZIP files, and is described in: http://www.w3.org/TR/PNG/#D-CRCAppendix. The link also provides an implementation example in C.

An array of size equal to the number of atoms is used to store the *identity* of each atom, and this is used in subsequent steps.

As well as assigning a value to each atom for subsequent use, each non-hydrogen atom is recorded in a datastructure with the following properties:

 hash code (32-bit signed integer)

 iteration (integer)

 atom list (array of integers)

For these initial atom assignments, the iteration is set to 0, and the atom list is an array with a single value, that being the index of the atom.

Note that duplicate hash codes *are* retained in the fingerprint list, because for the 0th iteration, entries with duplicate hash codes have a different *atom list*, which is important for subsequent steps.

If the requested fingerprint diameter is 0 (i.e. ECFP_0), the algorithm stops at this point.

#### Propagation

Once the initial atom identities are established, some number of iterations are performed. The most commonly used iteration count is 3, which gives rise to the ECFP_6 and FCFP_6 fingerprints. Each iteration generates zero-or-more additional hash codes. Tiny molecules may not generate any new fingerprints in iterations 2 or 3, i.e. the results for ECFP_2 or ECFP_4 may not be any different than what would have been obtained for ECFP_6 fingerprints.

The following process is repeated for each iteration, i.e. 1, 2 or 3 times.

A new *identity* array is defined, and for each non-hydrogen atom, a new identity value is calculated in the following way:

An array of pairs is defined, with one entry for each non-hydrogen neighbor. The values for each of these pairs are defined as [*bond*,*identity*], i.e.

 literal bond order (0, 1, 2, 3, …) or 15 if the bond is aromatic

 the identity of the neighbor atom from the previous iteration

These pairs are then sorted literally, first number first, e.g.

 [1,1000] < [2,-500]

 [1,-500] < [1,1000]

This list of pairs is then prefixed by the current iteration, and the current atom identity from the previous iteration. For example, performing iteration 1 on an atom with a previous identity of 200, and neighbour pairs of [2,-500] and [1,1000] would result in a sequence of [1,200,1,1000,2,-500].

These values are hashed into a CRC32 in the following way:

As a final addendum, if the atom has a *tetrahedral rubric* array associated with it, then an additional byte may be fed into the CRC32 hashing sequence. At the beginning of the overall fingerprint calculation process, each atom is assigned a *chirality* flag that is set to false. At each iteration, if the flag is still set to false, and there is a rubric array, then a determination is done: the rubric array of [A,B,C,D] is substituted for the *atom identity* (from the previous iteration) for each of its neighbours. Any neighbour that is a hydrogen atom (explicit or explicit) is given a value of 0. If any two values of this array are the same, then this step is skipped.

The new array of [a,b,c,d] is examined to determine how many *swaps* are necessary to order the array from lowest to highest. If the number of swaps is *even*, 1 is appended to the CRC32 sequence, otherwise 2 is appended. The *chirality* flag is set for the atom, so that it will not be further annotated in subsequent iterations.

Once the new identities are calculated for each heavy atom, the algorithm iterates over these atoms and creates a new fingerprint *proposal* for each one:

 hash code (newly computed for this iteration)

 iteration number (1, 2 or 3)

 atom list

 The last parameter, *atom list*, is a breadth first growth corresponding to the iteration. The initial fingerprints that were generated at iteration 0 defined this to contain a single atom index. Fingerprint proposals for iteration 1 contain the starting atom and all their neighbors; for iteration 2, the neighbors’ neighbors are included, etc. The atom lists may be cached for efficiency purposes or recomputed. They should also be sorted and not contain duplicates, e.g. if atom 10 has neighbors 5, 7 and 12, the atom list for the proposed fingerprint at iteration 1 would be [5],[7],[10],[12].

Once the proposed fingerprint is defined, the existing list of fingerprints is searched: if the sorted unique list of atoms matches any of the fingerprints already in the list, then only one of them may be retained:

 If the existing fingerprint has an earlier iteration number, discard the new one; else

 replace the existing fingerprint if the new one has a lower hash code.

This is done in order to reduce the degree of redundancy. Once the propagation steps are complete, the final output is the list of retained hash codes (signed 32-bit integers), sorted, with duplicates removed. These lists of integers are used for all of the similarity metrics described in this work.

### Similarity

*TB Mobile* allows the user to draw or paste a chemical structure as a query molecule, which causes the main compound display list to be sorted by most-to-least similar to the provided reference structure. Version 2 of the app has been updated to use the ECFP_6 fingerprints described in this work. Similarity is evaluated by computing the Tanimoto coefficient [58]. The calculation is done using the raw list of unique 32-bit hash codes, rather than folding into packed bitmasks.

### Clustering

The algorithm behind the visual clustering interface in *TB Mobile* performs a simple 2D embedding of a collection of molecules, then dynamically repositions them based on predefined tethers. The input parameters consist of a selection of compounds, and a central reference. For performance and display space purposes, the number of compounds is reduced by keeping the 50 most similar compounds to the reference, as determined by Tanimoto coefficient. The remaining compounds are converted into a graph by ensuring that each compound has an edge to each of the 10 most similar other compounds in the remaining set. If the resulting graph has multiple components, all components other than that which contains the reference are discarded. Once this process is complete, any compounds that are contained in the user-provided collection are added to the set, and tethered in the same way (i.e. they are always a part of the display, and are never pruned out).

Initial placement is done in a greedy fashion: the reference compound is placed at (0,0). The tether graph is walked in a breadth-first fashion, and for each bracket, the candidates are ordered by their average similarity to already placed compounds. Each is placed roughly by sampling positions that minimize the distance to tethered neighbors, but does not permit overlap, assuming that each node has a fixed radius. Once the initial placement is complete, the layout is shown to the user, and a background thread iteratively adjusts individual nodes to optimize a scoring term that encourages closeness tethered atoms vs. repulsion of non-tethered atoms. This is conceptually analogous to a forcefield, except with arbitrary terms tailored to aesthetic positioning.

### Target prediction

We updated *TB Mobile* with 60 new compounds and data (Additional file 1: Table S1) so the app now contains 805 compounds, (as of March 2014). There are 96 unique targets, for which 53 have 2 or more known binders in the dataset. The target/compound distribution is shown in Additional file 2: Table S2. We have evaluated the app with an additional set of 20 compounds (Additional file 3: Table S3). First they were all drawn in the *Mobile Molecular DataSheet* (MMDS) app and copied into the *TB Mobile* app (an example of app-to-app communication). Molecules can also be drawn within the *TB Mobile* app itself. The similarity searching component was used to rank the content in *TB Mobile* of molecules with known targets (Additional file 4: Figure S1-20). We have used this as an example of inferring potential targets and compared this to the published data for these molecules (Table 1). It should be noted that such data is far from definitive as these published compounds have not been tested versus all *Mtb* targets and it is possible the same compound may be active against more than one target. We generated screenshots for the top compounds (Additional file 4: Figure S1-20) and output the Bayesian scores into MMDS (Table 1).

**Table 1:** 20 compounds used to evaluate the app and target and predictions

**Molecule**	**Name**	**Published target and reference**	**Top target with similarity search**	**Bayesian target**	**Bayesian target**
				**Prediction with vers. 1 data (Bayesian-derived scores)**	**Prediction with vers. 2 data (Bayesian-derived scores)**
	Mathew cpd 1	Rv2150c FtsZ* [59]	InhA	Rv3790 DprE1 (0.35) Rv3423c Alr (0.57) Rv0206c MmpL3 (0.25)	Rv3791 DprE2 (0.09) Rv3790 DprE1 (0.16) Rv0005 GyrB (0.03) Rv3423c Alr (0.56) Rv0206c MmpL3 (0.01)
	Khan C-1	ATP synthase [60]	FabH	Rv1885c (0.002) Rv2150c FtsZ (0.08)	Rv2150c FtsZ (0.07)
	Khan C-2	ATP synthase [60]	KasB	Rv2150c FtsZ (0.06) Rv3423c Alr (0.11)	Rv2150c FtsZ (0.04) Rv3423c Alr (0.01)
	Khan C-3	ATP synthase [60]	KasB	Rv2150c FtsZ (0.09) Rv3423c Alr (0.08)	Rv2150c FtsZ (0.07) Rv3423c Alr (0.06)
	Khan C-4	ATP synthase [60]	InhA	Rv1885c (0.25) Rv3790 DprE1 (0.24)	Rv1885c (0.24) Rv3791 DprE2 (0.15) Rv3790 DprE1 (0.08)
	Khan C-5	ATP synthase [60]	KasB	Rv2150c FtsZ (0.14)	Rv2150c FtsZ (0.12)
	Khan C-6	ATP synthase [60]	KasB	Rv2150c FtsZ (0.09)	Rv2150c FtsZ (0.08)
	Vasudevan CymA	Rv 3596c ClpC1* [61]	PtpB	Rv2150c FtsZ (0.009) Rv2155c MurD (0.006) Rv2964 PurU (0.006)	Rv2150c FtsZ (0.01) Rv2155c MurD (0.007) Rv2964 PurU (0.007)
	Vasudevan CymA1	Rv 3596c ClpC1* [61]	PtpB	Rv2150c FtsZ (0.08)	Rv2150c FtsZ (0.08)
	Gao Domiphen	Rv 3582c IspD [62]	InhA	Rv2150c FtsZ (0.17) Rv1484 InhA (0.29) Rv3423c Alr (0.33) Rv0129c FbpC (1) Rv3794 EmbA (0.16)	Rv2150c FtsZ (0.15) Rv2780 Ald (0.35) Rv3791 DprE2 (0.008) Rv3790 DprE1 (0.03) Rv1484 InhA (0.17) Rv3423c Alr (0.34) Rv0129c FbpC (1) Rv3794 EmbA (0.15)
	Kale cpd 23	Rv0005 GyrB* [63]	AroD **(GyrB 4**^**th**^**)**	Rv2150c FtsZ (0.59) Rv3423c Alr (0.04) Rv3248c SahH (0.02)	Rv2150c FtsZ (0.53) Rv3791 DprE2 (0.06) Rv3790 DprE1 (0.13) **Rv0005 GyrB (0.19)** Rv3800c Pks13 (0.02) Rv3248c SahH (0.001)
	Pauli zinc 09137707	Rv1484 InhA* [64]	**InhA**	Rv2150c FtsZ (0.44) Rv3790 DprE1 (0.13) **Rv1484 InhA (0.37)** Rv3423c Alr (0.18)	Rv2150c FtsZ (0.39) Rv2780 Ald (0.46) Rv3791 DprE2 (0.03) Rv3790 DprE1 (0.15) **Rv1484 InhA (0.009)** Rv0005 GyrB (0.11) Rv3423c Alr (0.14) Rv3800c Pks13 (0.09)
	Pauli zinc 12509636	Rv1484 InhA* [64]	Kas B **(InhA 2**^**nd**^**and 3**^**rd**^**)**	No prediction	Rv3791 DprE2 (0.009)
	Pauli zinc 02931014	InhA		Rv2150c FtsZ (0.31)	Rv2150c FtsZ (0.27), Rv2780 Ald (0.55), Rv3790 DprE1 (0.03), Rv0005 GyrB (0.04)
	Pauli zinc 02931014	Rv1484 InhA* [64]	**InhA**	Rv2150c FtsZ (0.31)	Rv2150c FtsZ (0.27) Rv2780 Ald (0.55) Rv3790 DprE1 (0.03) Rv0005 GyrB (0.04)
	Wang cpd 4	Acetohydroxyacid synthase [65]	InhA	Rv1885c (0.07) Rv2155c MurD (0.11) Rv2964 PurU (0.11) Rv3790 DprE1 (0.09) Rv1484 InhA (1)	Rv1885c (0.07) Rv2155c MurD (0.11) Rv2964 PurU (0.11) Rv3790 DprE1 (0.03) Rv3791 DprE2 (0.16) Rv1484 InhA (1)
	Wang cpd 5	Acetohydroxyacid synthase [65]	Dxs1	Rv2150c FtsZ (0.4)	Rv2150c FtsZ (0.3) Rv3790 DprE1 (0.05)
	Wang cpd 7	Acetohydroxyacid synthase [65]	AroD	Rv2150c FtsZ (0.36) Rv2155c MurD (0.03) Rv2964 PurU (0.03) Rv3790 DprE1 (0.09)	Rv2150c FtsZ (0.32) Rv2155c MurD (0.03) Rv2964 PurU (0.03) Rv3790 DprE1 (0.12) Rv3791 DprE2 (0.11)
	Wang cpd 15	Acetohydroxyacid synthase [65]	Rv1885c	Rv1885c (1), Rv3790 DprE1 (0.18)	Rv1885c (1), Rv3790 DprE1 (0.18), Rv3790 DprE1 (0.11)
	Li cpd 4	Rv0548cMenB [66]	Glf	Rv1885c (0.26) Rv2150c FtsZ (0.13)	Rv1885c (0.26) Rv2150c FtsZ (0.13) Rv2780 Ald (0.06)
	Li cpd 5	Rv0548cMenB [66]	Glf	Rv1885c (0.22) Rv2150c FtsZ (0.13) Rv2155c MurD (0.06) Rv2964 PurU (0.06)	Rv1885c (0.22) Rv2150c FtsZ (0.13) Rv2780 Ald (0.07) Rv2155c MurD (0.06) Rv2964 PurU (0.06)

(*targets in TB mobile version 2.0, Targets in Bold are correctly predicted based on published data).

In order to provide a guide for whether a proposed compound is likely to be active against any of these targets, a simple modified Bayesian approach is used. For each listed target with at least two binders, it is first assumed that all of the molecules in the collection that do not indicate this as one of their targets are inactive. For each of the 805 constituent molecular structures, the ECFP_6 fingerprints are calculated. For each molecule, a score is determined using the Laplacian-modified naïve Bayesian classified described by Jenkins et al., among others [50],[51],[67]. The estimation score is a sum of log values, which can be transformed into a probability or used to classify as true or false based on a selected threshold.

**Figure 1:**
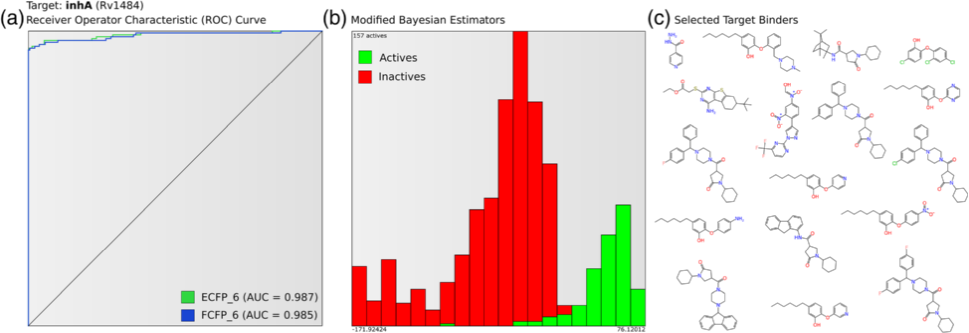
Predictions for the InhA target: (a) the ROC curve with ECFP_6 and FCFP_6 fingerprints; (b) modified Bayesian estimators for active and inactive compounds; (c) structures of selected binders.

The score for each molecule is calculated in a leave-one-out fashion, i.e. its own contribution is excluded from the list of known fingerprints and activities. The score is a sum of log values of probabilities, with arbitrary upper and lower limits. For these a threshold can be selected, above which a molecule is predicted to be active against the target. By varying the threshold between the upper and lower bounds, it is also possible to create a receiver-operator-characteristic (ROC) curve, by determining the true/false positives and negatives for each threshold. Figure 1 shows response metrics for the InhA target which has 157 compounds targeting it. We have generated similar analyses for other targets in *TB Mobile* (Additional file 5: Table S4).

The ROC curve in Figure 1a shows the response for both the ECFP_6 fingerprints described in this work, as well as the FCFP_6 variant. At the same time as we submitted the ECFP fingerprint capabilities to the CDK project, we also made available the FCFP equivalent, which is identical except that it initially describes each atom by whether it is an H-bond donor/acceptor, positive/negative charge center, aromatic and/or halogen. For the *TB Mobile* app, we use only the ECFP variant. Figure 1b shows the Bayesian estimator scores, binned at intervals to produce a bar chart. As can be seen, there is little overlap between the scores for the known binders and the remaining compounds in the collection, with the caveat that the small dataset is biased by a number of recurring structural motifs, some of which are shown in Figure 1c.

When the mobile app considers a user-proposed compound for possible activity against a given target, the modified Bayesian score is calculated in the same way. If the resulting score falls within the range of the ROC plot, i.e. if the score were to be used as a threshold then the confusion matrix would report at least one false positive or false negative, then the probability of activity is expressed as a value between 0 and 1, depending where it falls between the lower and upper bounds. If the score lies outside of the range of the ROC plot, it is set to 0 or 1.

This prediction method is essentially a normalized similarity metric, for which proposed compounds are ranked favorably if they are similar to known target binders. This approach compliments the sorting of molecules in *TB Mobile* by similarity described herein.

### Interoperability

**Figure 2:**
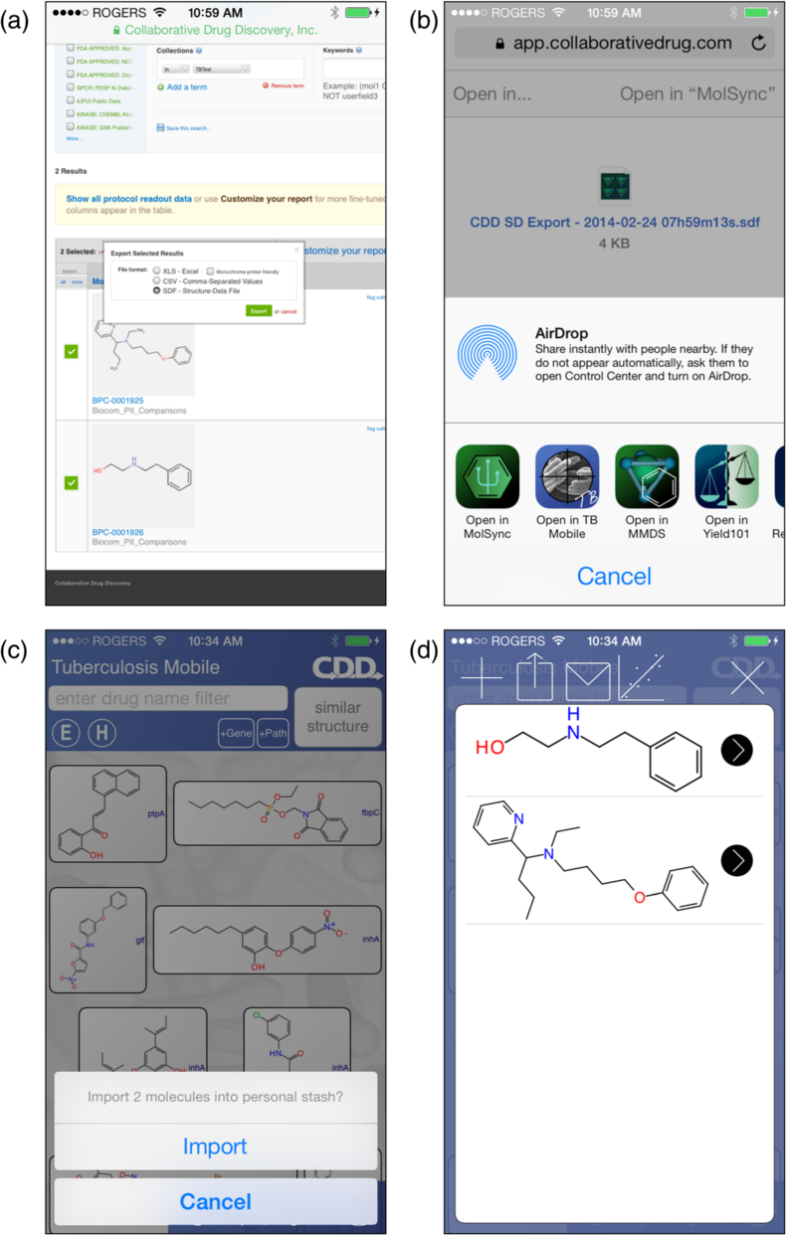
**Importing compounds from the web: (a) setting up an SDfile download with CDD Vault; (b) selecting the** ***TB Mobile*** **app as the destination; (c) molecule import on launch; (d) the personal stash, with imported structures.**

The *TB Mobile* app supports a number of ways of moving data into and out of the app, which is in keeping with the mobile app interoperability features we have described previously [68],[69]. Other than providing a built-in structure editor [70] and the ability to read structure formats from the clipboard, the importing capability is offered by registering the app as a receiver for a several common structure formats, such as MDL Molfile and SDfile. As shown in Figure 2, this can be used to connect the app with content downloaded from the mobile browser. The same mechanism applies to downloaded email attachments, files from hosting apps such as *Dropbox*, and the launching of content dynamically generated by other apps.

**Figure 3:**
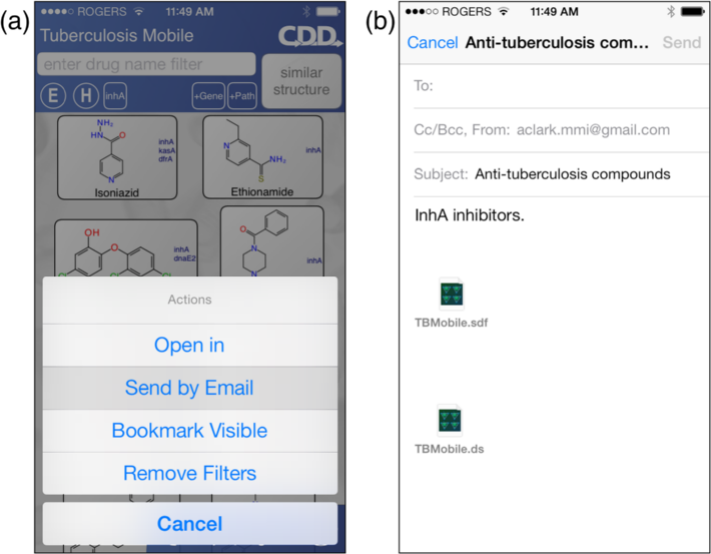
Sending selected compounds by email: (a) initiating the transmission for structures shown onscreen; (b) writing the email, with prepackaged attachments.

**Figure 4:**
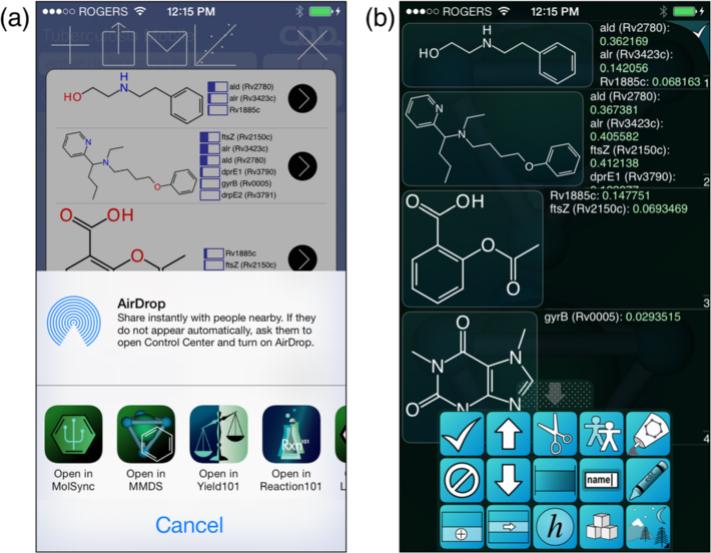
**Exporting the personal collection: (a) initiating the export, the molecules in the compound collection havebar icons representing predicted targets using the Bayesian models with extended connectivity fingerprints.** The top icons (from left to right) represent adding molecules – which can be drawn in the app, drawn with other apps on the device, pasted in from elsewhere or there is the option to remove all structures. The second icon allows the contents of the molecule stash to be opened in other apps on the device. The third icon allows you to email the contents of the molecule stash and the fourth icon generated target predictions.The arrow icon aligned with each molecule allows the molecule to be either copied to the clipboard, transferred to the main window on the app or the structure can be edited or deleted; **(b)** importing the datasheet into MMDS, with predictions included.

Exporting content can be achieved in several ways. From within the detail view for any given compound, the molecular structure can be copied to the clipboard or launched into another app. From the main menu, it is possible to initiate an outgoing email with attachments that contain structures for all of the compounds currently being displayed. Figure 3 shows an example where all structures that bind the InhA target (a) are shown, and the outgoing email (b) in progress. The datasheet attachments are included automatically by the app. The personal collection of compounds can also be exported by launching with other apps, as shown in Figure 4a. If target predictions have been made for these compounds, then they will be included as additional fields, which can be viewed and manipulated using other apps such as MMDS, shown in Figure 4b.

**Figure 5:**
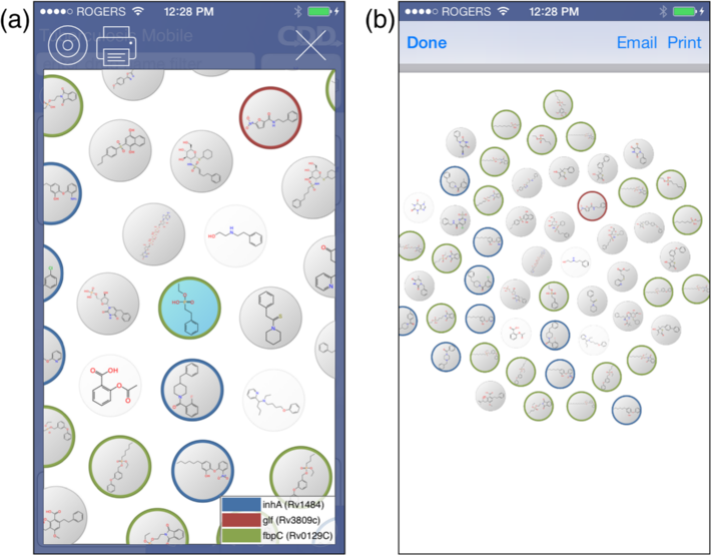
**Exporting presentation graphics for a cluster.** An example of clustering with the known targets of molecules highlighted in different colors. This is enabled by selecting the icon on the top left and choosing the desired targets. Molecules from the collection are shown with a white circle, molecules from the app have a grey background and compounds of interest for clustering have a blue background. The cluster image can be expanded or contracted with a pinching motion on the screen. and molecules can be moved which causes the network clusters to reconfigure. The cluster image can be output and printed as a PDF. **(a)** the interactive display; **(b)** previewing the PDF file, prior to sending by email or printing.

The clustering feature can be used to create presentation quality graphics, as shown in Figure 5. On request, the app will create a PDF document with a single page representing the cluster, which is previewed onscreen. From there it can be sent as an email attachment, or printed directly from the app, if there is a printer configured and accessible.

### TB mobile app software application

We have previously described the functions and applications of the *TB Mobile* app in detail [30] which uses molecule structures and their targets. In version 2.0 we have updated the aesthetics and we still enable the original features such as scrolling through molecules and similarity searching but now we based the similarity comparisons on our ECFP_6 implementation. Most similar compounds are listed first (from top left to bottom right) in the app.

## Results

### Fingerprints comparison with published data

Several collections of TB related compounds have been previously used for generating Bayesian Models by us. For two TB *in vitro* screening datasets, with activity expressed as true or false, a simple Bayesian model was constructed, using a popular Laplacian-corrected variant implemented in this study for *TB Mobile*[50],[51],[67]. The activity for each compound was predicted using leave-one-out. Using a grade of cutoff thresholds, an ROC plot is created, by plotting false negatives against false positives. This enabled comparisons to the previously published ROC values for the two datasets using FCFP_6 descriptors, noting that in these published models several interpretable descriptors were also included. It also enabled us to compare how ECFP_6 and FCFP_6 descriptors perform for the same datasets.

We observed that for the models generated in this study and previously published, the ROC values were comparable (Table 2). In addition there was no appreciable difference between the performance of ECFP_6 and FCFP_6 descriptors for the same datasets.

**Table 2:** Testing the fingerprints used in TB Mobile 2.0

**Dataset**	**Leave one out ROC published**	**Reference**	**Leave one out ROC in this study**
Combined model (5304 molecules) ECFP_6 fingerprints	N/A	N/A	0.77
Combined model (5304 molecules) FCFP_6 fingerprints	0.71	[47]	0.77
MLSMR dual event model (2273 molecules) and ECFP_6 fingerprints	N/A	N/A	0.84
MLSMR dual event model (2273 molecules) and FCFP_6 fingerprints	0.86	[46]	0.83

Previously published *Mtb* HTS datasets were used for Bayesian model building. Published studies used FCFP_6 descriptors and several interpretable descriptors were also included [46],[47].

### Dataset curation

**Figure 6:**
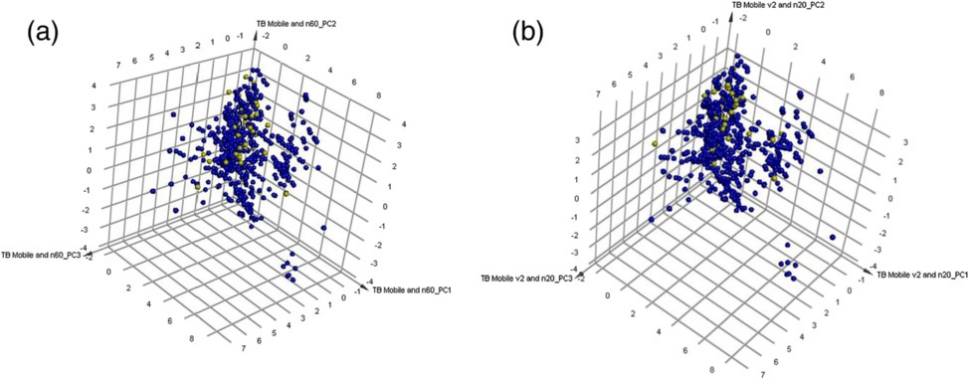
**Principal component analysis. a**. Using the 745 compounds in TB Mobile version 1 and the 60 additional compounds added in TB Mobile 2. 3 Principal Components represent 88.4% of the variance. **b**. Using the 805 compounds in TB Mobile version 2 and the 20 additional compounds used to evaluate it. 3 Principal Components represent 88.4% of the variance.

An additional 60 molecules with target related information from the literature (Additional file 1: Table S1) were curated for use in CDD and *TB Mobile*. These compounds were assessed using principal component analysis (PCA) using Discovery Studio with the interpretable descriptors chosen previously (AlogP, molecular weight, number of rotatable bonds, number of rings, number of aromatic rings, number of hydrogen bond acceptors, number of hydrogen bond donors, and molecular fractional polar surface area) to assess their overlap in chemical space (Figure 6a). The new compounds appear to be within the existing chemical space of the original dataset.

### TB mobile version 2.0

As with the previously described version 1 of *TB Mobile*[30] the app first organizes its data, then displays the main screen. We will now describe and focus on the newly added features.

### Predicting targets for new compounds

We have curated an additional set of 20 compounds (Additional file 3: Table S3) which were scored with version 2 of the app (Table 1). Only a few of the targets represented in this set were in TB mobile e.g. FtsZ, InhA, ClpC1, GyrB. Interestingly the one compound targeting FtsZ was predicted based on similarity ranking in the app to target InhA (Additional file 4: Figure S1), while the Bayesian predictions suggested multiple targets (Table 1). While bedaquiline is known to target ATP synthase [10],[11] it was not shown to be similar in *TB Mobile* to the ATP synthase targeting compounds in the test set (Additional file 4: Figure S2-7). The very large macrocyclic compounds were not predicted by similarity ranking or Bayesian approaches in the app (Additional file 4: Figure S8-9). These compounds do however look similar to PtpB compounds for which there are 3 examples in *TB Mobile* (Additional file 5: Table S4e). The GyrB inhibitor was correctly ranked in the app (Additional file 4: Figure S10) and was one of the targets selected with the Bayesian model approach (Table 1). The three InhA compounds (Additional file 4: Figure S11-13) were well predicted based on ranking in the app with 2 out of the three being ranked as the top targets, while one was selected also using the Bayesian approach alongside several other targets (Table 1). A set of acetohydroxyacid synthase and MenB inhibitors were predicted to be similar to several targets (Additional file 4: Figure S14-S20), based on searching *TB Mobile* and using Bayesian approaches (Table 1). The comparison of Bayesian models generated with version 1 data (N = 745 molecules) and version 2 data suggests that some targets are added with the Bayesian approach (see Kale cpd 23, (Table 1) for which GyrB is predicted in version 2 but not version 1). These 20 compounds were also assessed using PCA. Most of the compounds were within the space of the 805 compounds although there were several that were outside or on the edge of this chemistry space such as the macrocyclic compounds (Figure 6b). This evaluation set illustrates how difficult it is identify targets for new compounds. Many contain substructures that are present in compounds known to bind other targets. In addition limitations of the approach are clear if the target is not represented in the dataset to begin with. This approach also illustrates an opportunity to try to identify or design compounds targeting multiple *Mtb* targets by using features shared by several targets.

## Discussion and conclusions

Our goal in creating *TB Mobile*[30] was to make this potentially useful drug discovery data from CDD available in a form accessible to scientists in general and provide a novel way to predict potential targets. We have seen in recent years a clear development in apps that can be used in drug discovery or chemistry [71], which suggested to us that creating a mobile app would ensure we reached a much wider audience. This work also follows on from our efforts to make other types of science data more readily accessible such as green solvent data [72] and rare and neglected disease data [73]. *TB Mobile* was made freely available for iOS (iPhone, iPod, iPad) in 2012 and Android devices in 2013 and has been updated regularly. It has been downloaded nearly 2000 times to date and has been used by us elsewhere [33]. While our initial apps have focused on performing one or two functions we have proposed that apps can be used in workflows [68],[69]. With version 2 we have greatly expanded *TB Mobile* so that not only does it provide a look up of the molecules with known targets and other information, we can also load a library of molecules which can then be used for prediction. The prediction of target-molecules is enabled using extended connectivity fingerprints (ECFP_6) and a naïve Bayesian method and can in turn output the data.

This report now highlights a more valuable component of mobile app workflows to see if the compound had been previously identified by others, what the most similar molecules are and their known targets which could help in lead optimization. We also should highlight that there will be compounds that we are unable to predict with the Bayesian models in *TB Mobile* but they can be used for a similarity searching (Table 1, Additional file 4: Figure S1-S20). This may represent a useful way to identify compounds that may be too dissimilar to compounds in *TB Mobile*. Clearly the app was developed to suggest potential targets to assist researchers in identifying potential targets. But it is a prediction based on a relatively limited number of targets and their known ligands and should be used with some caution. We are not trying to predict affinity for the targets but to narrow down the potential number of targets for experimental verification.

In the process of curating data we have added an additional 18 targets/mechanisms as well as a number of additional compounds for other recent targets of interest such as MmpL3 which should help balance the bias towards targets that are over represented like InhA. We previously suggested the need to normalize the similarity search for the frequency of a target in the dataset which essentially we have achieved with the Bayesian method that builds models for the individual targets (Additional file 5: Table S4). These efforts to collate data for individual *Mtb* targets may in itself be useful for drug design purposes. The curation of the 20 molecule evaluation set could in turn be added back into the app so that it now includes the additional targets unique to this set.

Version 2 of *TB Mobile* expands on the delivery of high quality data by adding some advanced workflow capabilities, in a manner that is interactive and very accessible to scientists who are not experts in cheminformatics. As data regarding tuberculosis targets continues to be collated (e.g. 80 molecules are included here in total), we intend to release periodic updates of the app. Presently the source data is delivered within the app itself, and so additional data is made available by issuing updates through the iTunes AppStore. In the future we may further enhance *TB Mobile* so that it is capable of automatically adding new data from an online source, as it becomes available, which will ensure that the content is current.

The proof of concept for bringing together high quality cheminformatics and bioinformatics data and easy to use visualization on a mobile device has been carried out for the tuberculosis domain, since it is an area of high interest on account of the emergence of new strains of drug resistant bacteria and its massive toll on global public health. We can also use the app as a test case to develop such approaches on the desktop and enhance the CDD database. We are also actively looking into ways to bring this mobile app workflow to other domains that have a tradition of relatively nonproprietary data access, whether that be by creating a separate app for each subject, or looking for ways to deploy the app more generally.

We are further investigating ways to increase the platform independence of this product. Mobile devices are popular, and building a native app is the most effective way to provide a comprehensive and responsive user experience. The high cost of porting the product to every major device platform is prohibitive. Our development priority targets Apple’s iOS platform as the most important category since it has an overwhelming advantage in the western hemisphere, but the same cannot be said for emerging economies, which is particularly pertinent for a disease that has the most devastating effects on tropical nations. For this reason we are actively monitoring the evolution of cross-platform and pure-web technologies. In this case we may update the Android version content alone and not the features for the foreseeable future.

As we have previously used the molecules in *TB Mobile* to compare to other sets of molecules to assess how they compare to target space using PCA [44],[47], the addition of the additional compounds increases the number of targets covered. This updated dataset will be useful for future PCA comparisons like those described here (Figure 6a and 6b). Now that we have an open source version of the extended connectivity fingerprints and naïve Bayesian algorithm, we could incorporate other predictive models in *TB Mobile* such as those we have described to predict whole cell activity alongside predicting targets [32],[36],[37],[43]-[47],[49]. We could also extend the concept of *TB Mobile* to other diseases, for example diseases like malaria might be obvious examples where there is considerable recent data on screening that could benefit from target prediction methods to prioritize compounds.

## Competing interests

Sean Ekins is a consultant for Collaborative Drug Discovery Inc. Alex M. Clark is the founder of Molecular Materials Informatics, Inc., and developed all the apps described.

## Authors’ contributions

AMC developed TB Mobile, the algorithms, fingerprints and wrote the manuscript, SE came up with the idea for TB Mobile, performed all the evaluation of the app and wrote the manuscript. MS curated database links to the molecules used in TB Mobile and wrote the manuscript. All authors read and approved the final manuscript.

## Additional files

## Supplementary Material

Additional file 1: Table S1. The 60 compounds and targets added into *TB Mobile* version 2 are in Table S1. Click here for file

Additional file 2: Table S2. *Mtb*Target distribution in TB Mobile Vers.2.0. Click here for file

Additional file 3: Table S3. The test set of 20 compounds and their target and pathway details are shown in Table S3. Click here for file

Additional file 4: Figure S1-20. The results of the similarity searches for compounds in Table S3 are shown in Figures S1-20. The *TB Mobile* app is freely available from the Apple iTunes AppStore [40]. Click here for file

Additional file 5: Table S4. The Bayesian models for targets are shown in (a-e) and shows the target prediction charts and selected binders for targets with at least 3 examples. Click here for file
