# Supplementary material for: New target prediction and visualization tools incorporating open source molecular fingerprints for TB Mobile 2.0
Source: J Cheminform. 2014 Aug 4;6:38. doi: 10.1186/s13321-014-0038-2 (PMC4190048; doi:10.1186/s13321-014-0038-2)
Supplement: Additional file 2: Table S2. — MtbTarget distribution in TB Mobile Vers.2.0. [file s13321-014-0038-2-S2.docx]

**Additional file 2: Table S2. *Mtb*Target distribution in TB Mobile Vers.2.0**

| **Gene** | **Count** | **Gene** | **Count** |
| --- | --- | --- | --- |
| Rv0283 | 1 | Rv0678 | 1 |
| Rv1211 | 1 | Rv1685c | 1 |
| Rv1885c | 4 | Rv3160c | 1 |
| Rv3161c | 1 | TB27.3 (Rv0577) | 2 |
| ald (Rv2780) | 2 | alr (Rv3423c) | 8 |
| aroD (Rv2537c) | 14 | aspS (Rv2572c) | 1 |
| atpE (Rv1305) | 2 | blaC (Rv2068c) | 1 |
| clpB (Rv0384c) | 1 | clpC (Rv3596c) | 1 |
| cyp121 (Rv2276) | 2 | cyp130 (Rv1256c) | 2 |
| cyp51 (Rv0764c) | 2 | cysH (Rv2392) | 10 |
| cysS (Rv2130c) | 1 | dacB2 (Rv2911) | 1 |
| dapA (Rv2753c) | 12 | deaD (Rv1253) | 1 |
| def (Rv0429c) | 14 | dfrA (Rv2763c) | 3 |
| dinG (Rv1329c)" (count=1) | 1 | dlaT (Rv2215) | 2 |
| dnaA (Rv0001)" (count=1) | 1 | dnaB (Rv0058) | 1 |
| dnaE2 (Rv3370c)" (count=1) | 1 | dprE1 (Rv3790) | 8 |
| dprE2" (count=1) | 1 | drpE2 (Rv3791) | 2 |
| dxr (Rv2870c) | 1 | dxs1 (Rv2682C) | 29 |
| embA (Rv3794) | 2 | embB (Rv3795) | 1 |
| embC (Rv3793) | 1 | engA (Rv1713) | 1 |
| era (Rv2364c) | 1 | ethA (Rv3854c) | 1 |
| fabG (Rv0242c) | 2 | fabH (Rv0533) | 48 |
| fadD32 (Rv3801c) | 5 | fbpC (Rv0129C) | 21 |
| folP1 (Rv3608C) | 1 | folP2 (Rv1207) | 1 |
| frdA (Rv1552) | 1 | ftsZ (Rv2150c) | 3 |
| fusA1 (Rv0684) | 3 | fusA2 (Rv0120c) | 3 |
| glcB (Rv837c) | 1 | glf (Rv3809c) | 40 |
| glmU (Rv1018c) | 1 | guab2 (Rv3411) | 1 |
| gyrA (Rv0006) | 24 | gyrB (Rv0005) | 9 |
| ilvG (Rv1820) | 1 | infB (Rv2839c) | 1 |
| inhA (Rv1484) | 157 | kasA (Rv2245) | 9 |
| kasB (Rv2246) | 5 | ldtMt1 (Rv0116c) | 4 |
| ldtMt2 (Rv2518c) | 1 | lpd (Rv0462) | 5 |
| lppS (Rv2515c) | 1 | mbtA (Rv2384) | 95 |
| mca (Rv1082) | 27 | mfd (Rv1020) | 1 |
| mmpL3 (Rv0206c) | 15 | moeW (Rv2338c) | 1 |
| mshB (Rv1170) | 4 | murB (Rv0482) | 1 |
| murD (Rv2155c) | 2 | nadB (Rv1595) | 1 |
| ndhA (Rv0392c) | 1 | nrdR (Rv2718c) | 2 |
| pH Homeostasis | 5 | panC (Rv3602c) | 20 |
| pks13 (Rv3800c) | 3 | proteasome | 2 |
| ptpA (Rv2234) | 38 | ptpB (Rv0153c) | 3 |
| purU (Rv2964) | 2 | qcrB (Rv2196) | 5 |
| quinol oxidase | 1 | recG (Rv2973c) | 1 |
| rplC (Rv0701) | 2 | rplJ (Rv0651) | 3 |
| rpoB (Rv0667) | 4 | sahH (Rv3248c) | 2 |
| thiL (Rv2977c) | 106 | tlyA (Rv1694) | 2 |
| tuf (Rv0685) | 3 | uvrA (Rv1638) | 1 |
