# Supplementary material for: New target prediction and visualization tools incorporating open source molecular fingerprints for TB Mobile 2.0
Source: J Cheminform. 2014 Aug 4;6:38. doi: 10.1186/s13321-014-0038-2 (PMC4190048; doi:10.1186/s13321-014-0038-2)

Additional file 4: Figure S1. Mathew cpd 1 similarity search in TB mobile vers. 2.0. Query molecule = top right.


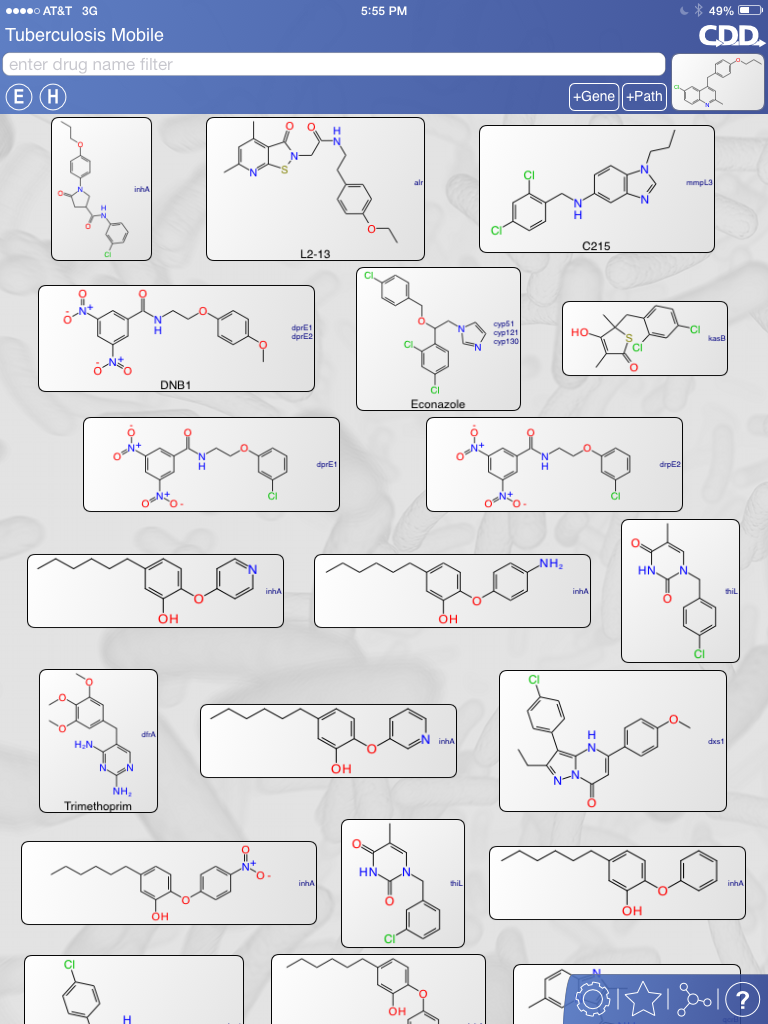


Figure S2. Khan C-1 similarity search in TB mobile vers. 2.0. Query molecule = top right.


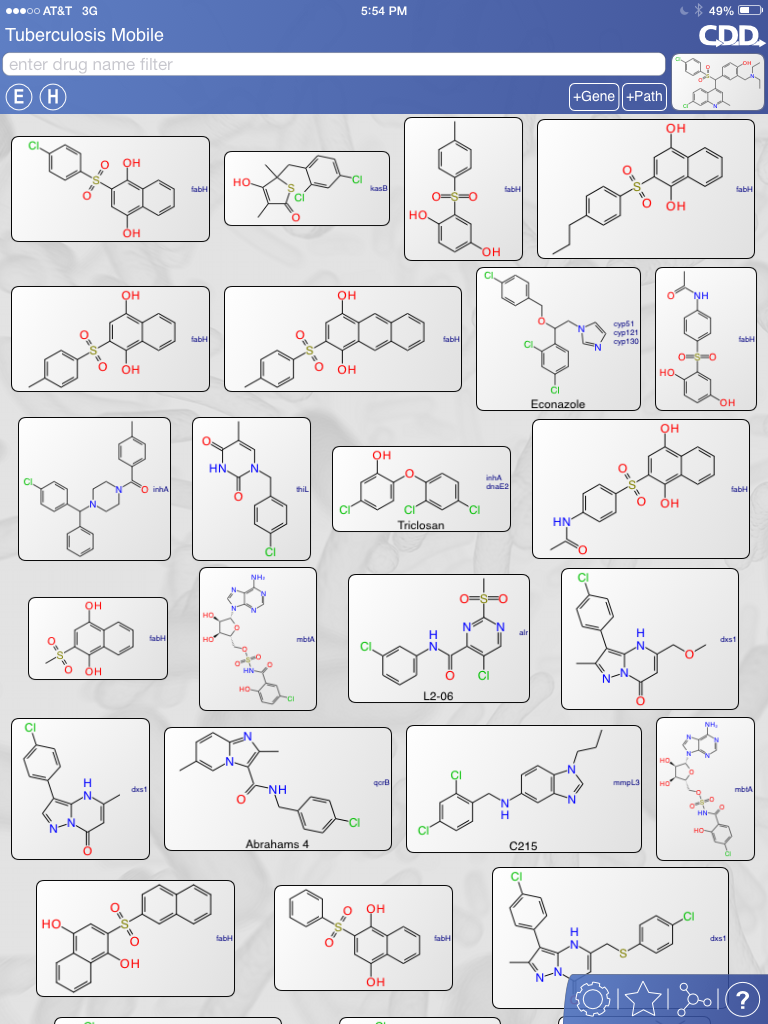


Figure S3. Khan C-2 similarity search in TB mobile vers. 2.0. Query molecule = top right.


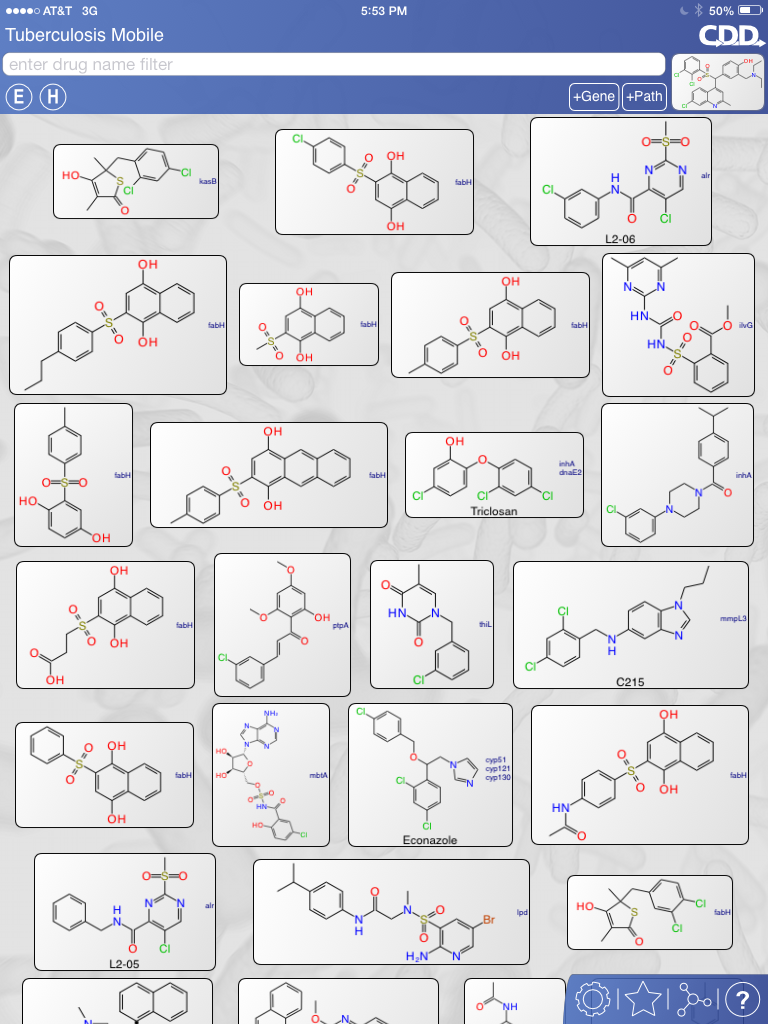


Figure S4. Khan C-3 similarity search in TB mobile vers. 2.0. Query molecule = top right.


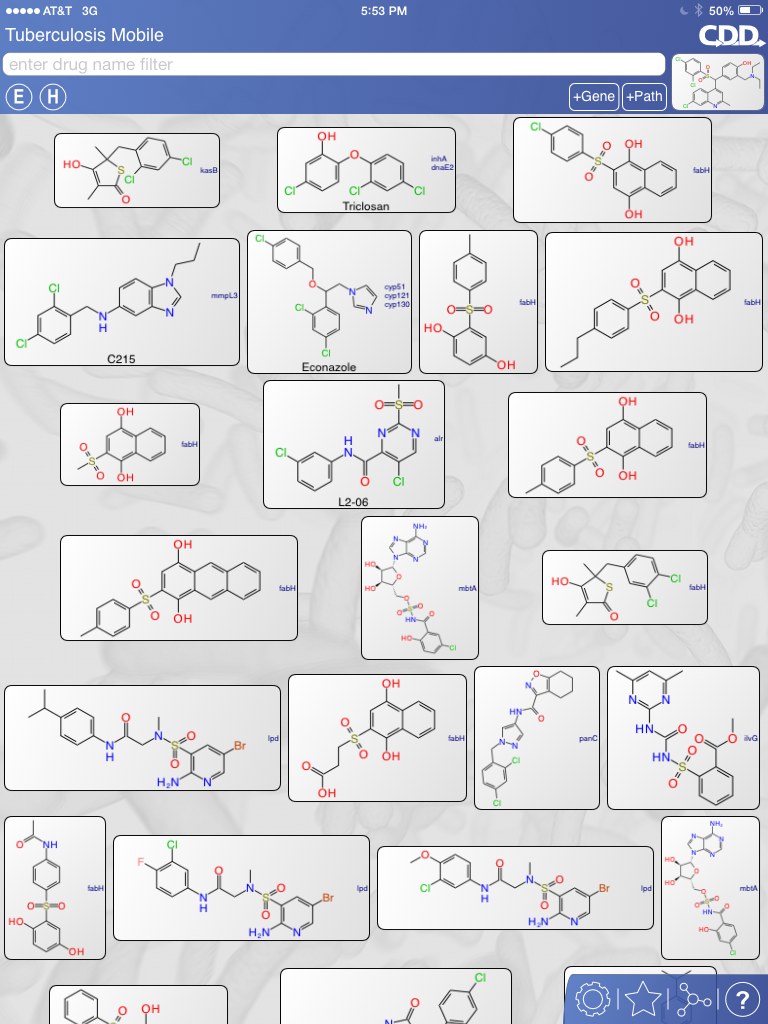


Figure S5. Khan C-4 similarity search in TB mobile vers. 2.0. Query molecule = top right.
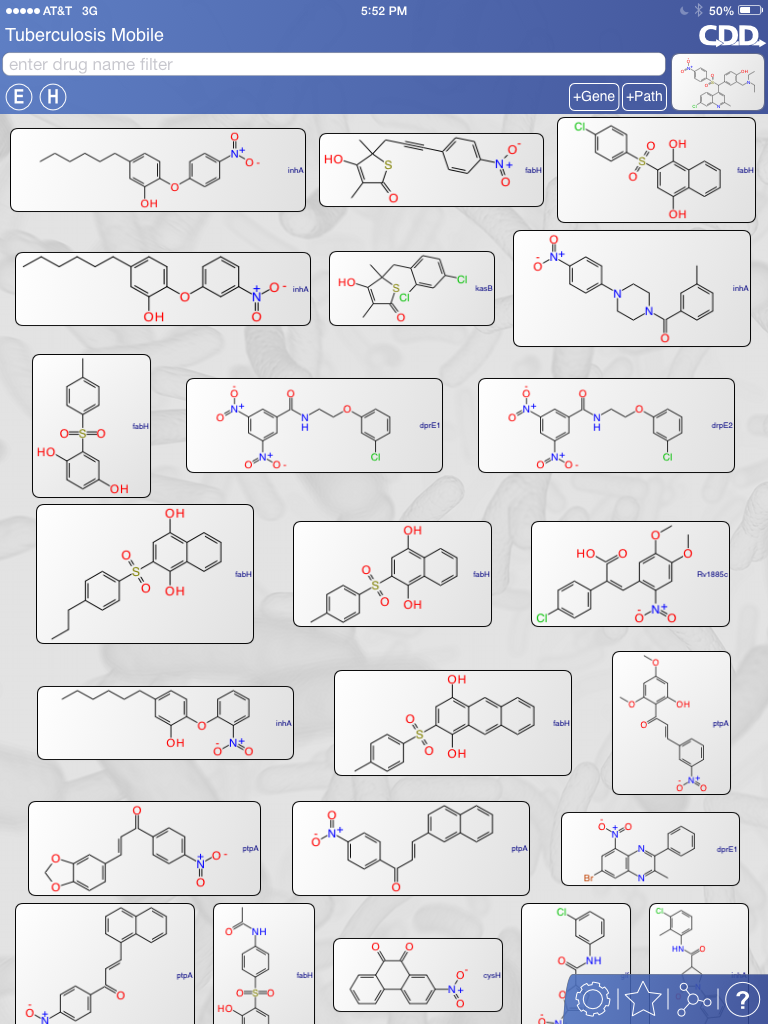


Figure S6. Khan C-5 similarity search in TB mobile vers. 2.0. Query molecule = top right.


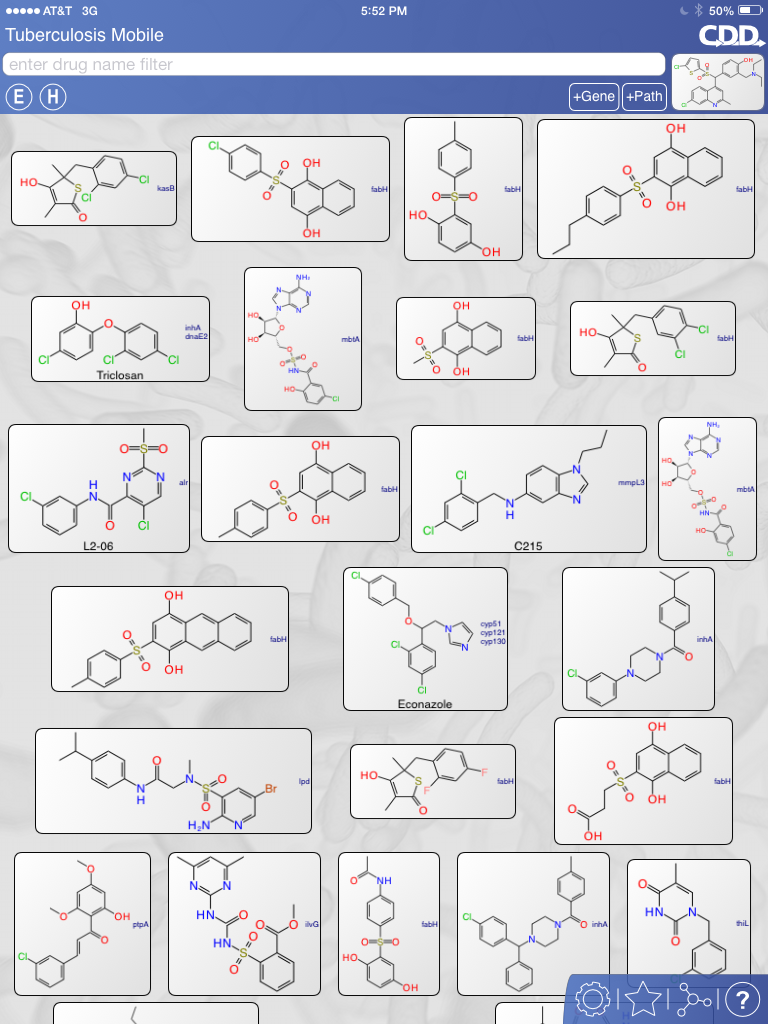


Figure S7. Khan C-6 similarity search in TB mobile vers. 2.0. Query molecule = top right.


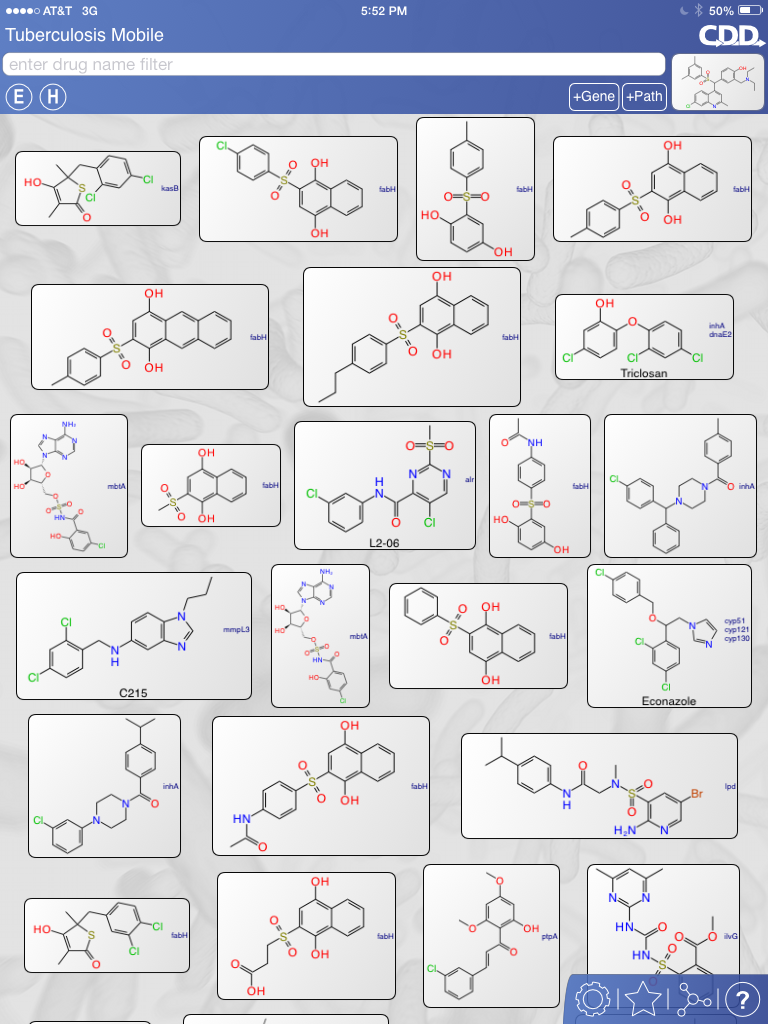


Figure S8. Vasudevan CymA similarity search in TB mobile vers. 2.0. Query molecule = top right.


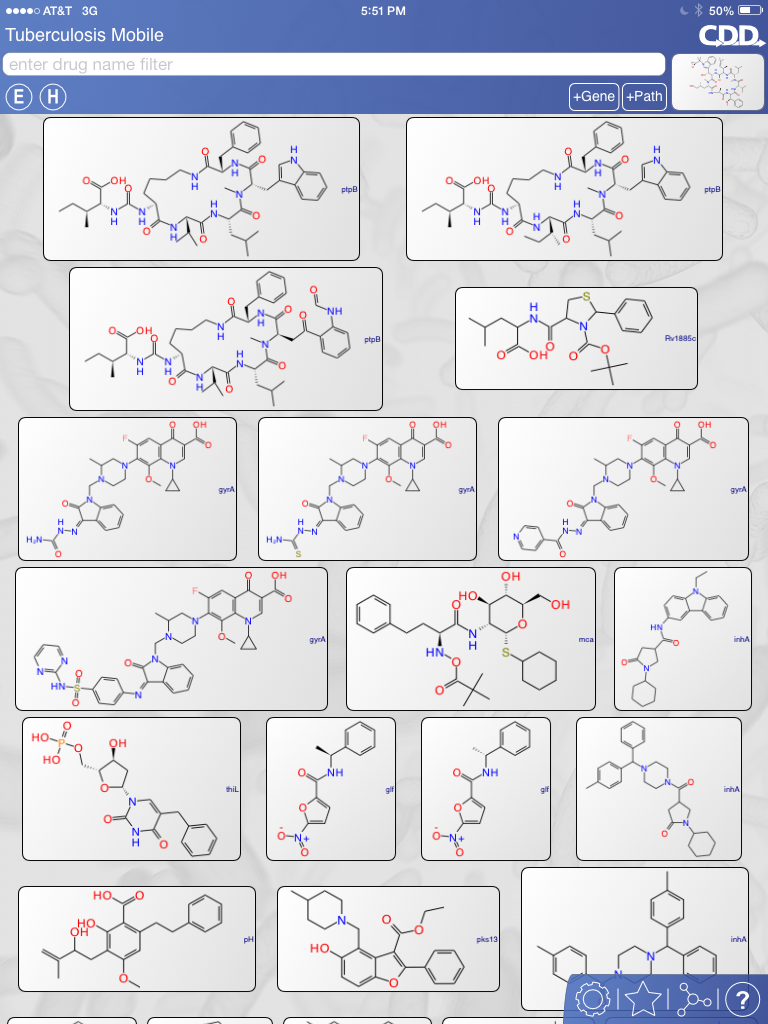


Figure S9. Vasudevan CymA1 similarity search in TB mobile vers. 2.0. Query molecule = top right.


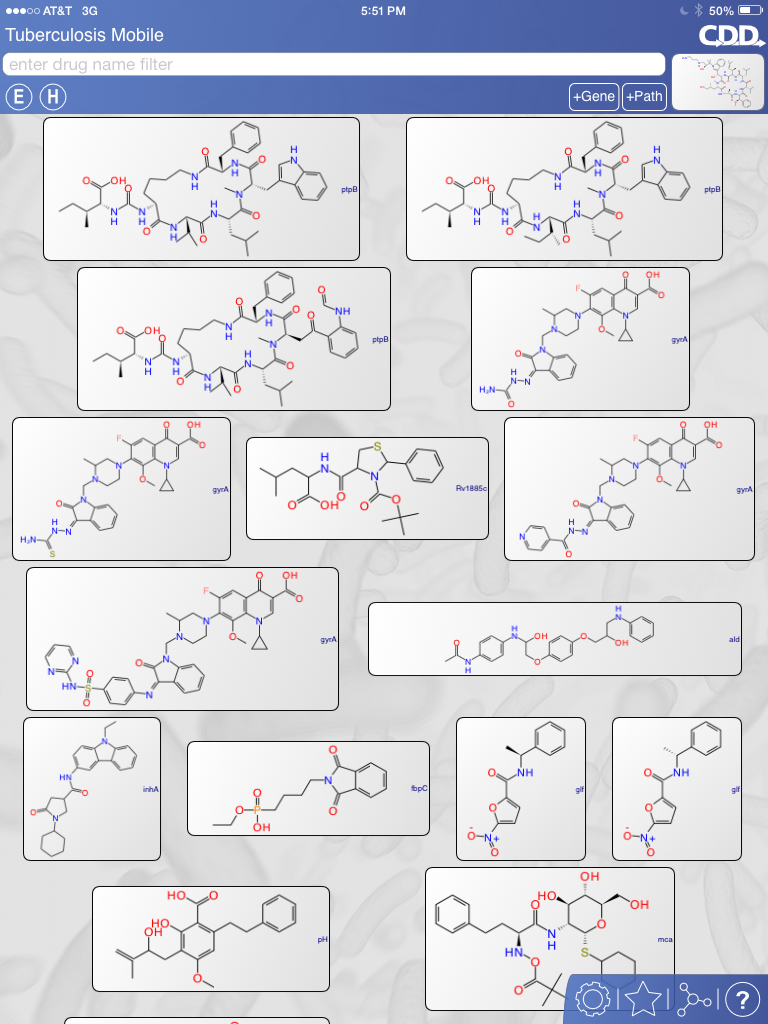


Figure S10. Gao Domiphen similarity search in TB mobile vers. 2.0. Query molecule = top right.


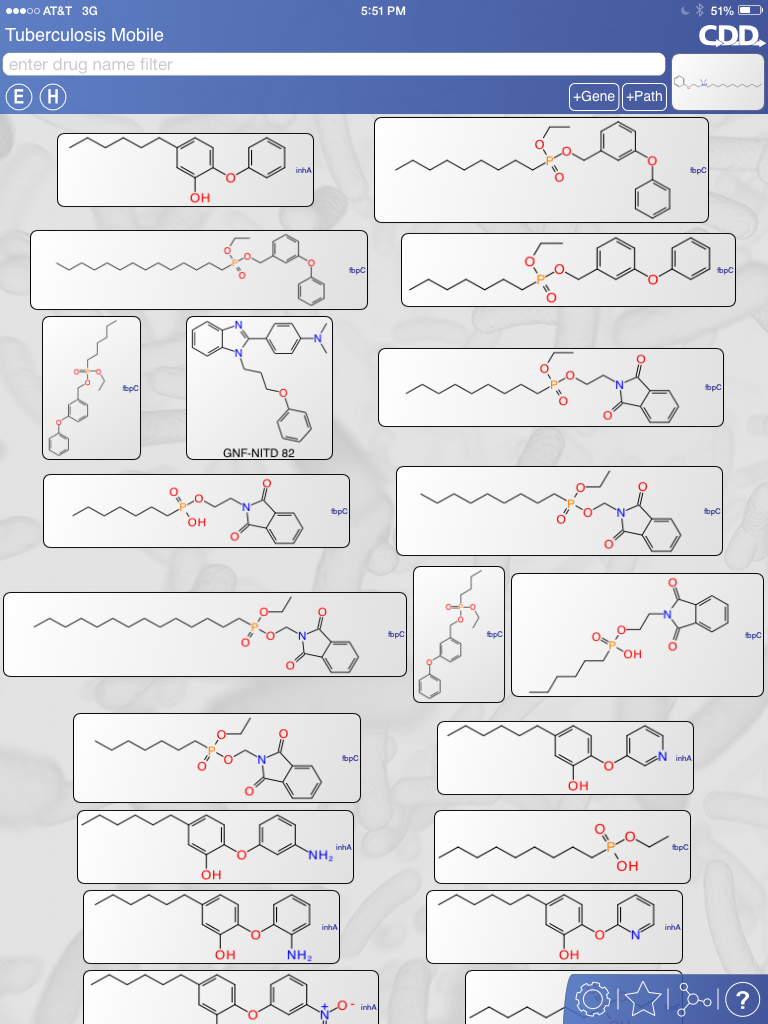


Figure S11 Kale cpd 23 similarity search in TB mobile vers. 2.0. Query molecule = top right.


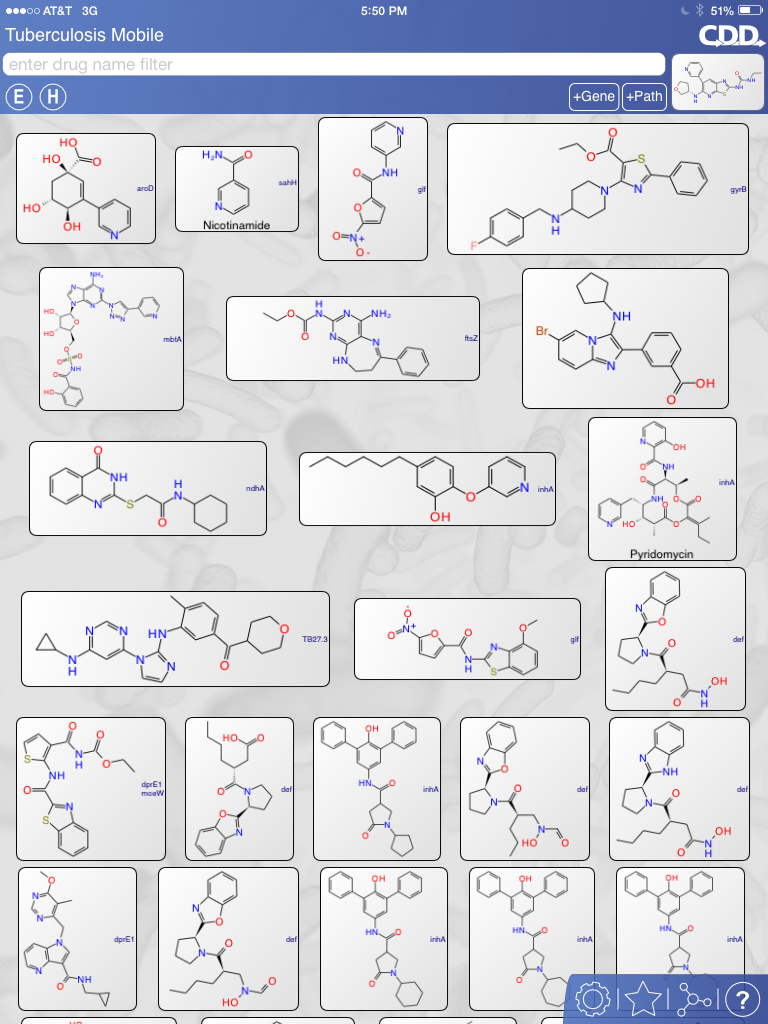


Figure S12. Pauli ZINC 09137707 similarity search in TB mobile vers. 2.0. Query molecule = top right.


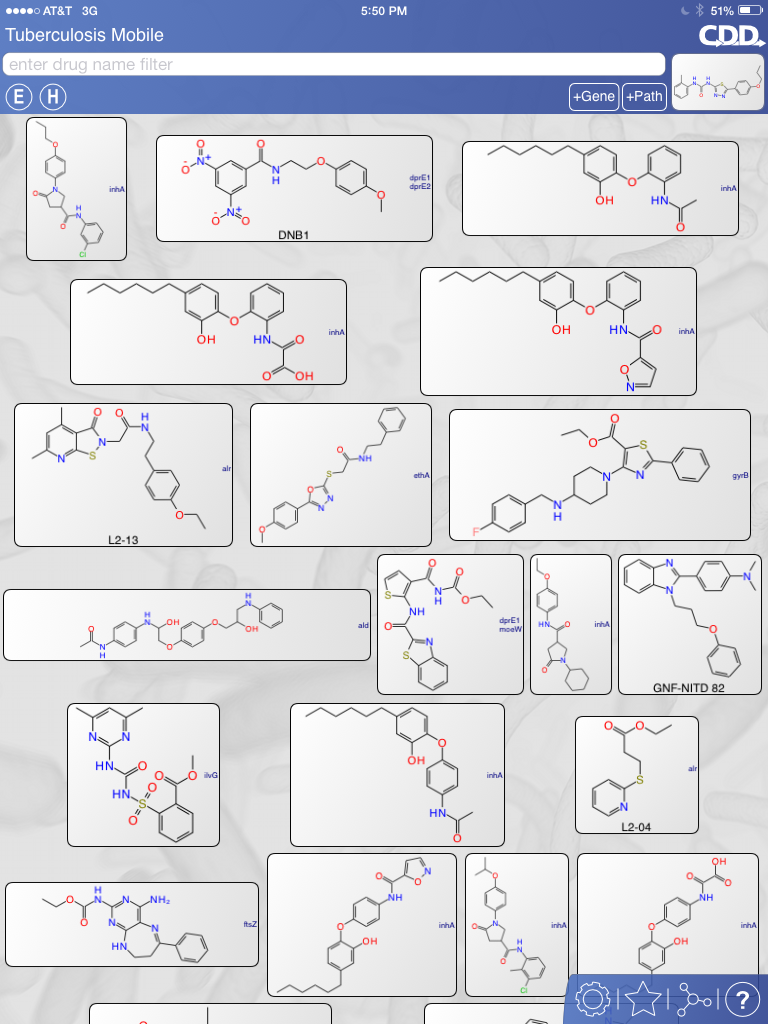


Figure S13. Pauli ZINC 12509636 similarity search in TB mobile vers. 2.0. Query molecule = top right.


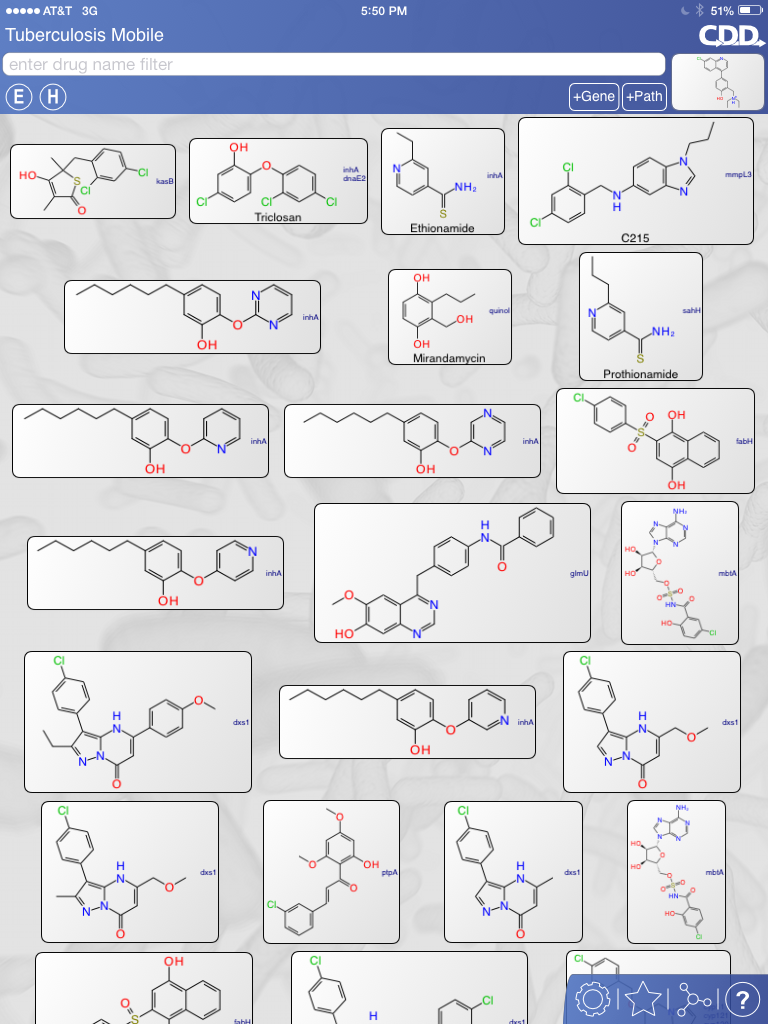


Figure S14. Pauli ZINC 02931014 similarity search in TB mobile vers. 2.0. Query molecule = top right.


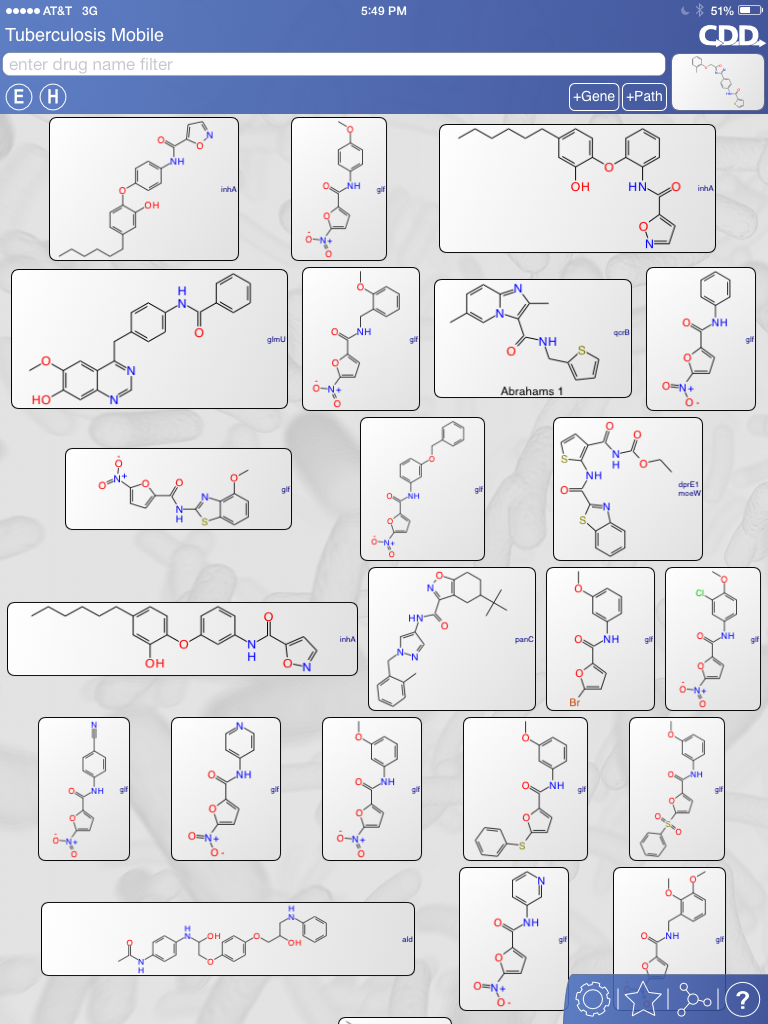


Figure S15. Wang cpd 4 similarity search in TB mobile vers. 2.0. Query molecule = top right.


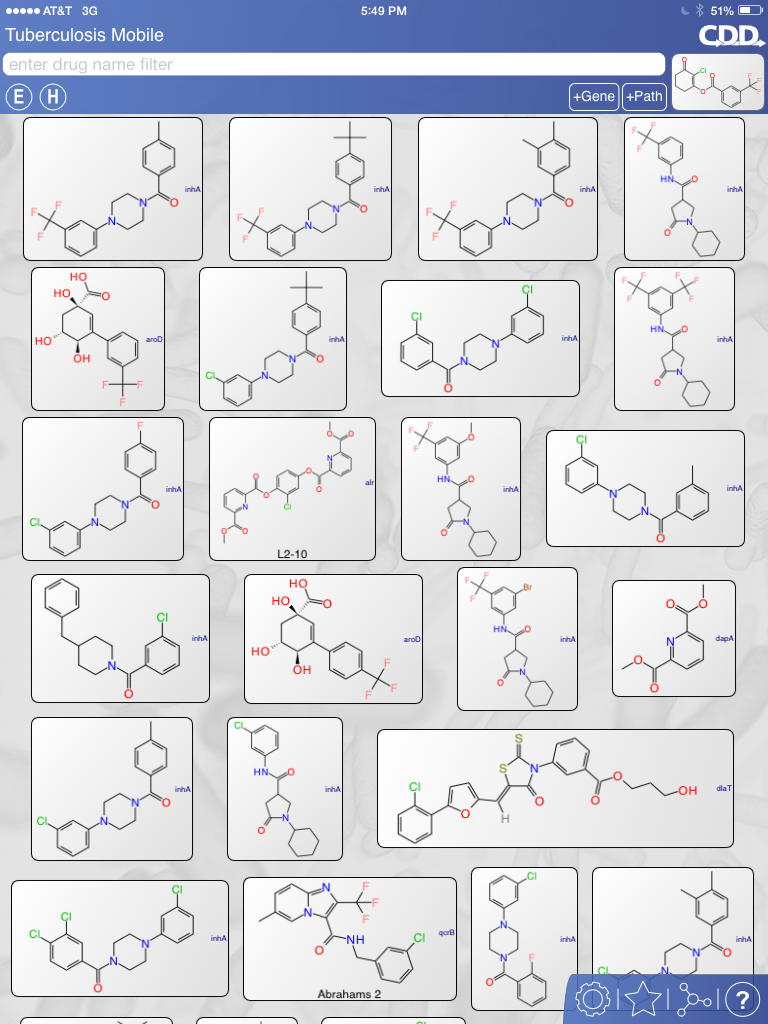


Figure S16. Wang cpd 5 similarity search in TB mobile vers. 2.0. Query molecule = top right.


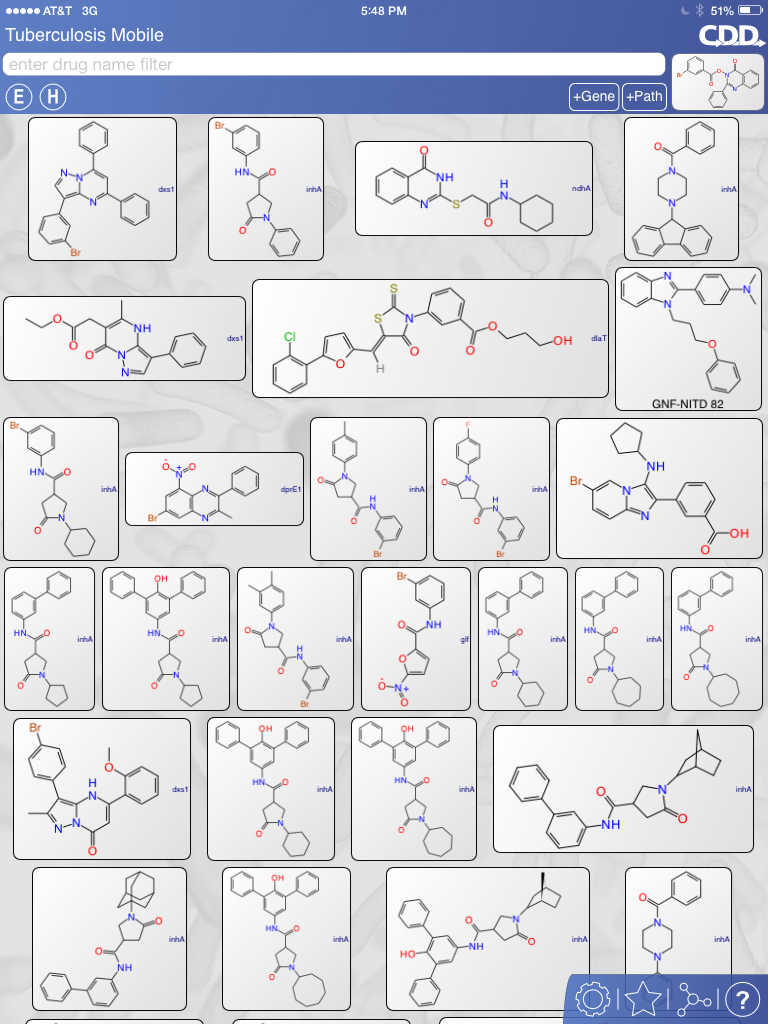


Figure S17. Wang cpd 7 similarity search in TB mobile vers. 2.0. Query molecule = top right.


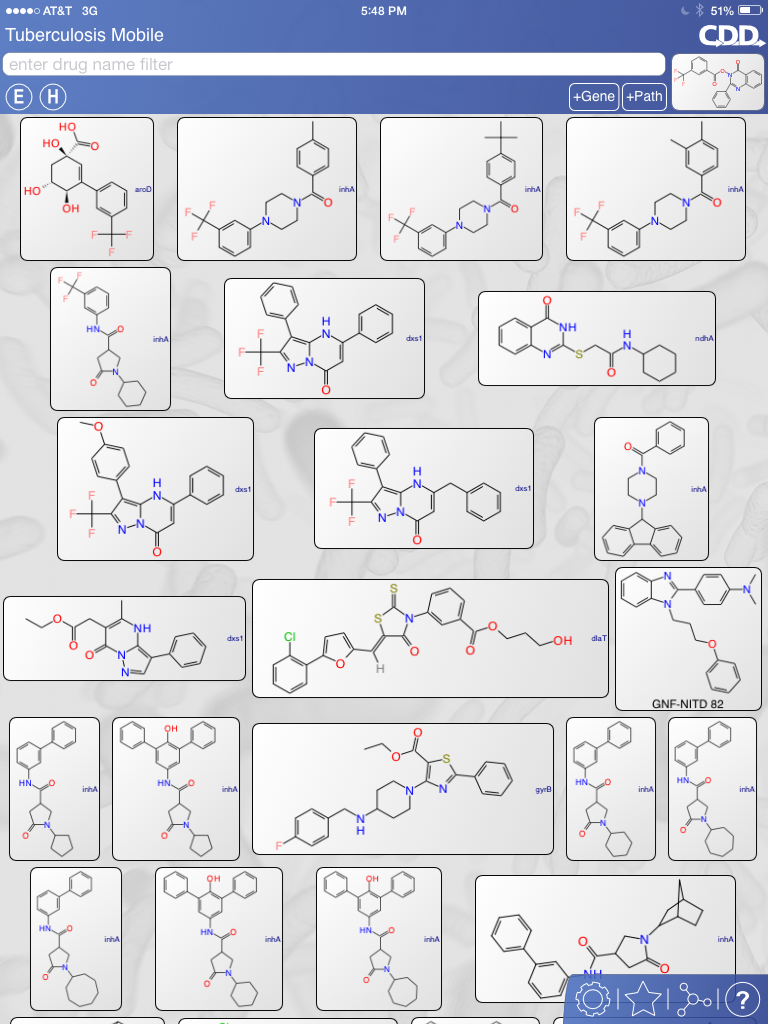


Figure S18. Wang cpd 15 similarity search in TB mobile vers. 2.0. Query molecule = top right.


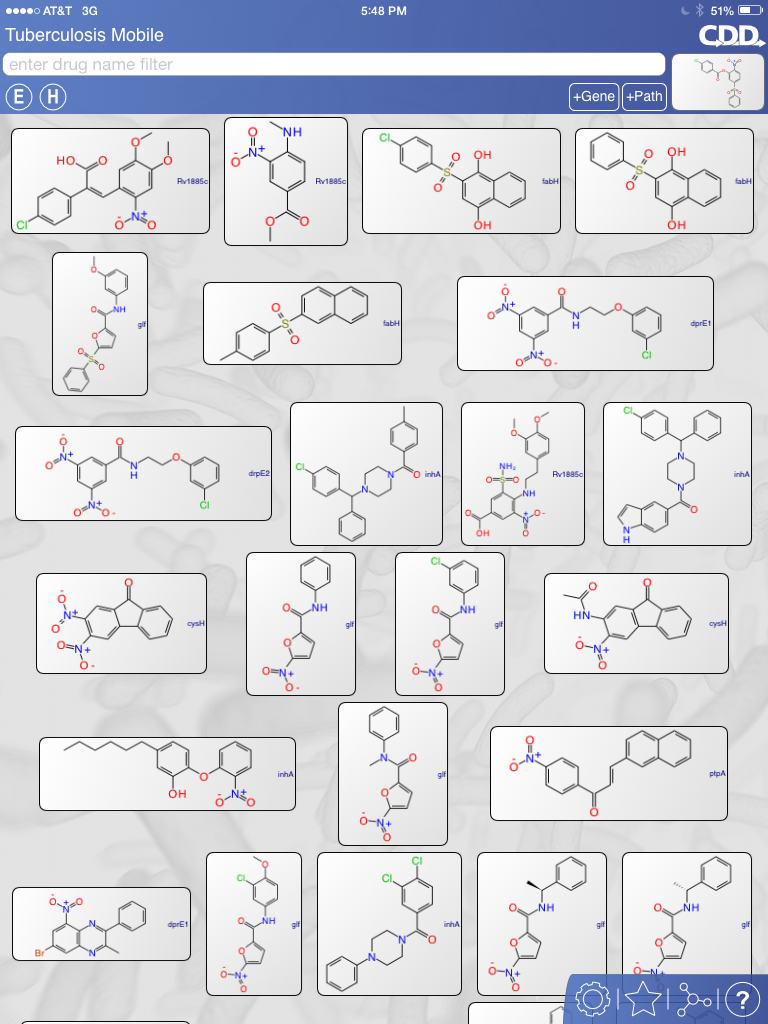


Figure S19. Li cpd 4 similarity search in TB mobile vers. 2.0. Query molecule = top right.


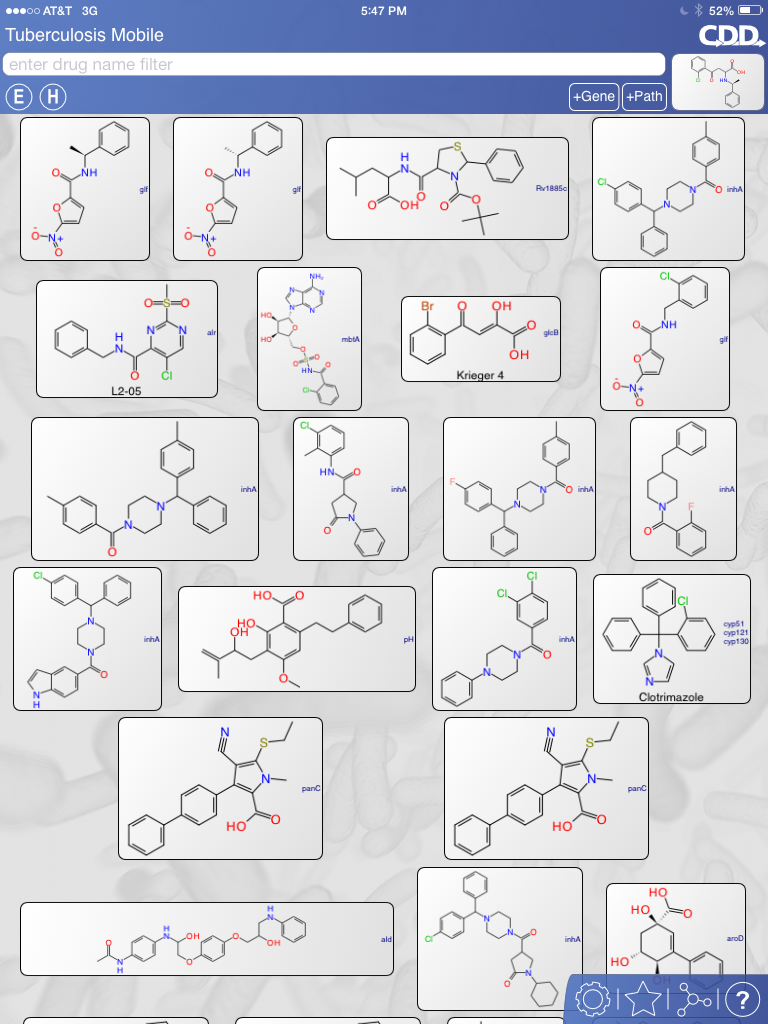


Figure S20. Li cpd 5 similarity search in TB mobile vers. 2.0. Query molecule = top right.


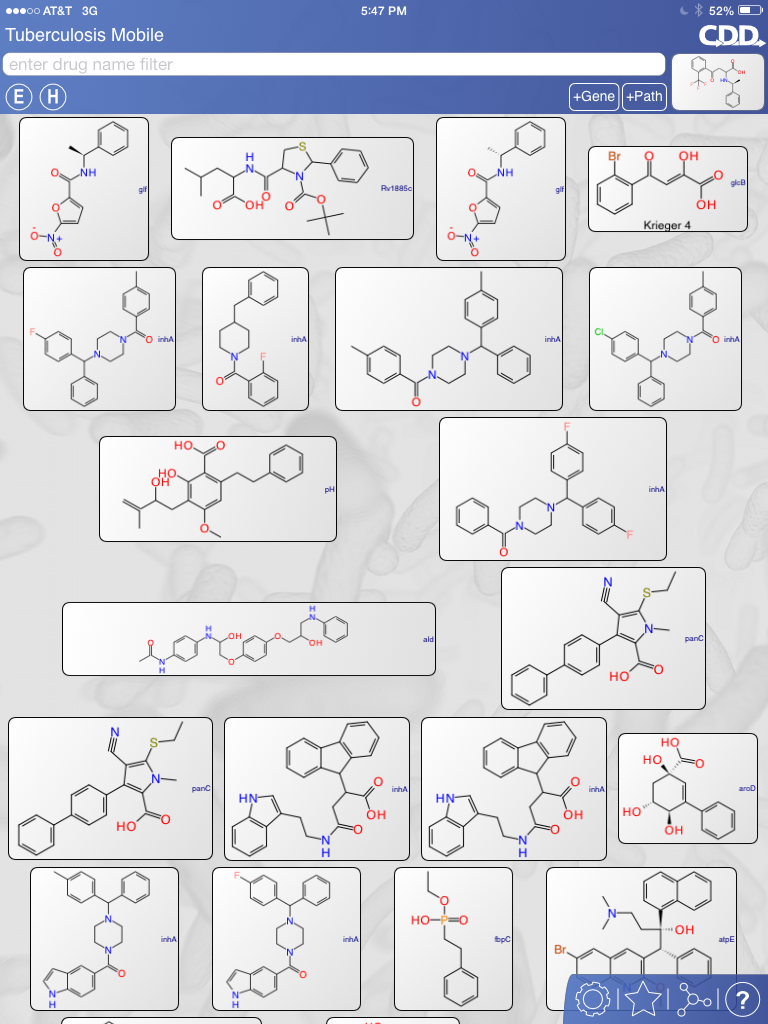

Supplement: Additional file 4: Figure S1-20. — The results of the similarity searches for compounds in Table S3 are shown in Figures S1-20. The TB Mobile app is freely available from the Apple iTunes AppStore [40]. [file s13321-014-0038-2-S4.docx]
