# Supplementary material for: New target prediction and visualization tools incorporating open source molecular fingerprints for TB Mobile 2.0
Source: J Cheminform. 2014 Aug 4;6:38. doi: 10.1186/s13321-014-0038-2 (PMC4190048; doi:10.1186/s13321-014-0038-2)

Bayesian inhA (Rv1484)

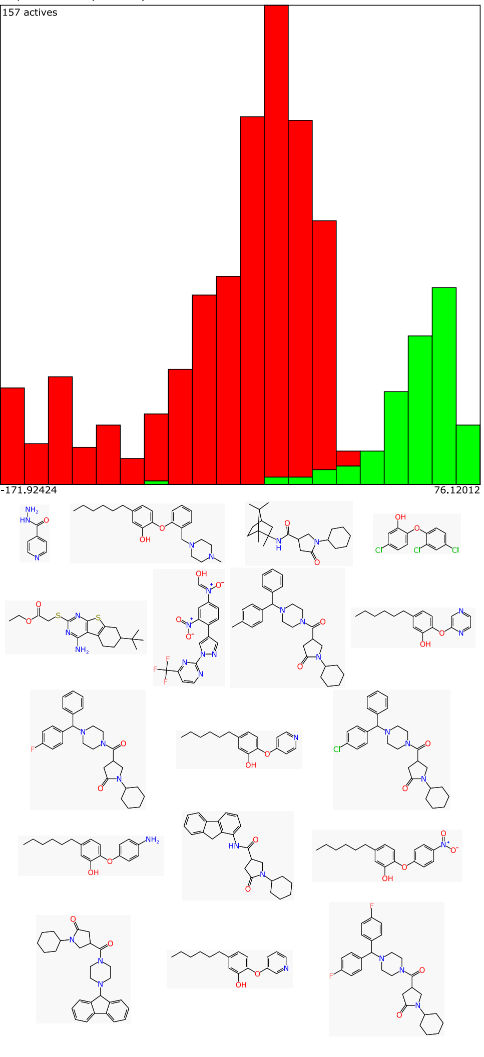

Bayesian mbtA (Rv2384)

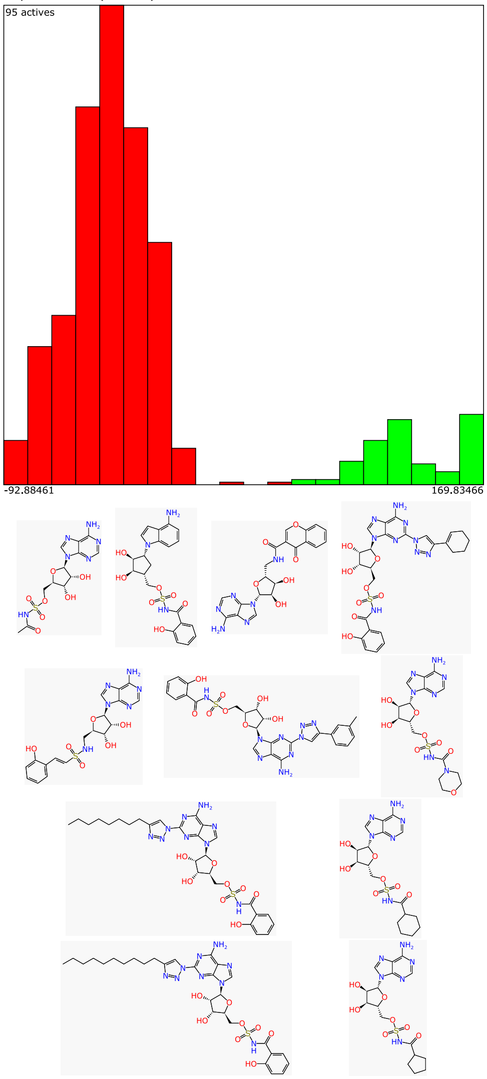

Bayesian mca (Rv1082)

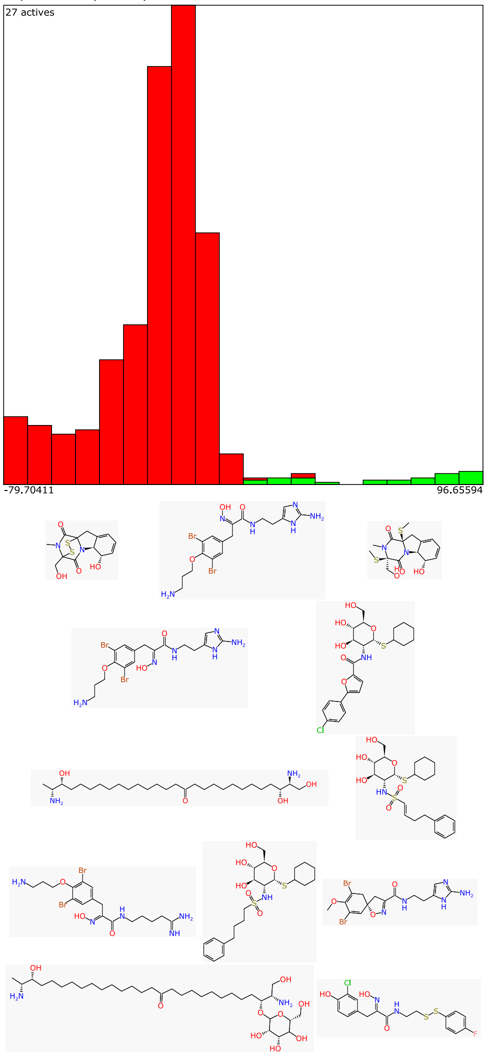

Bayesian panC (Rv3602c)

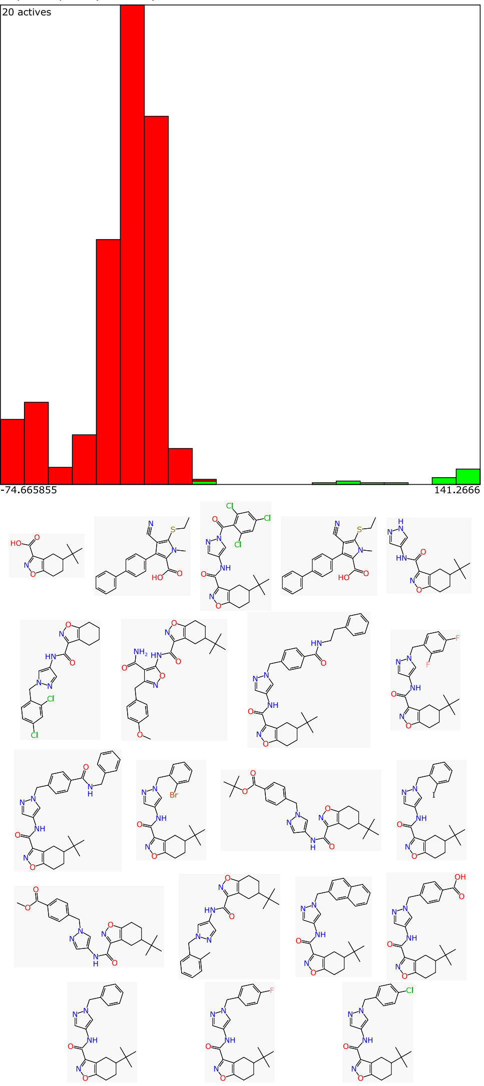

Bayesian ptpA (Rv2234)

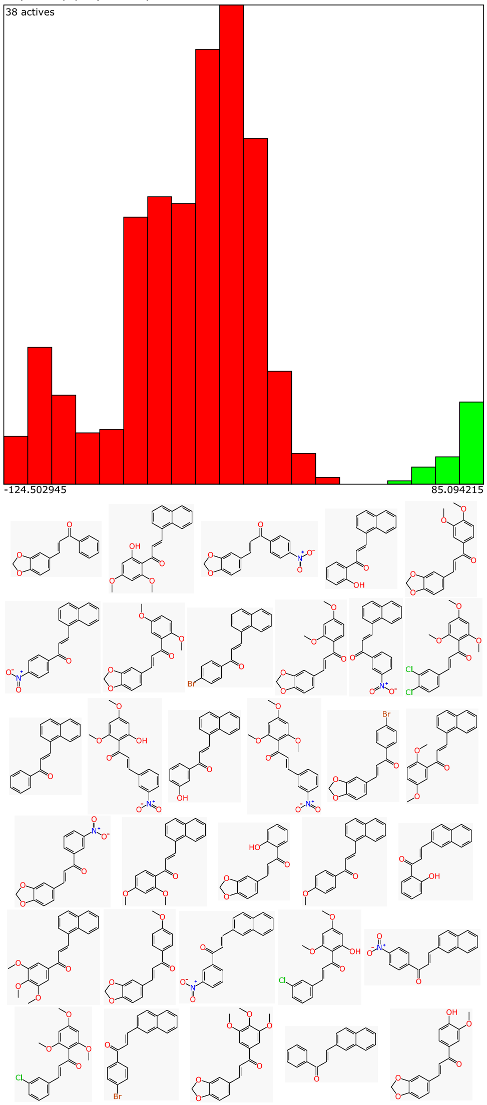

Bayesian thiL (Rv2977c)

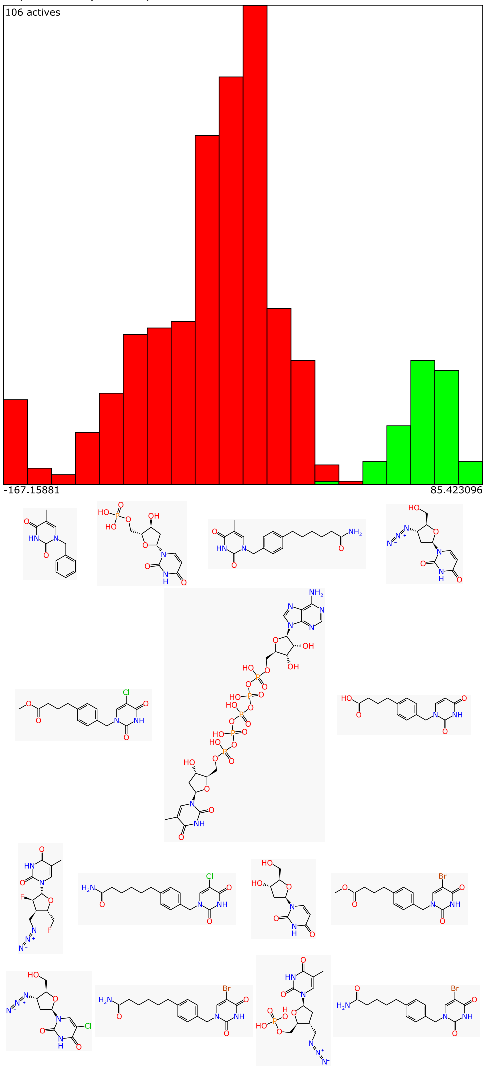

Supplement: Additional file 5: Table S4. — The Bayesian models for targets are shown in (a-e) and shows the target prediction charts and selected binders for targets with at least 3 examples. [file s13321-014-0038-2-S5.zip › 2034153920123298_add4b.pdf]
