# Supplementary figures and images for: New target prediction and visualization tools incorporating open source molecular fingerprints for TB Mobile 2.0
Source: J Cheminform. 2014 Aug 4;6:38. doi: 10.1186/s13321-014-0038-2 (PMC4190048; doi:10.1186/s13321-014-0038-2)

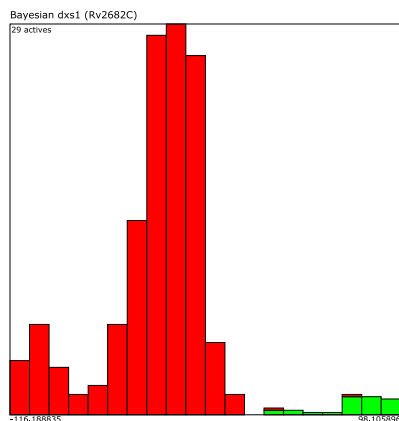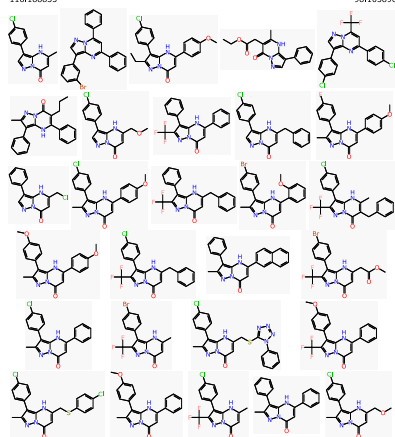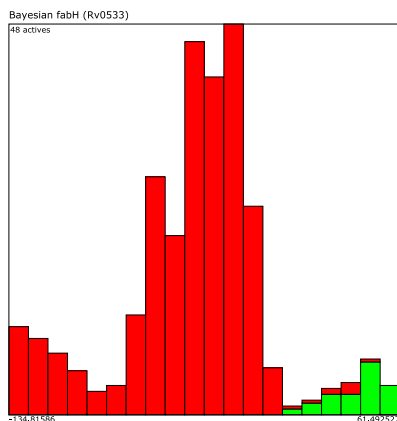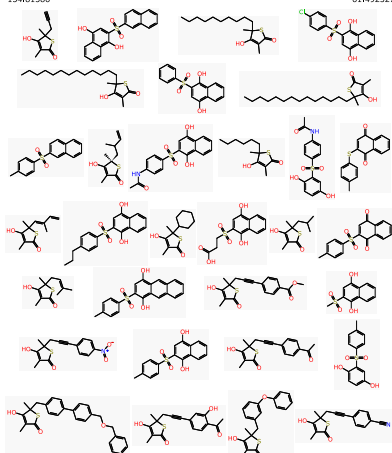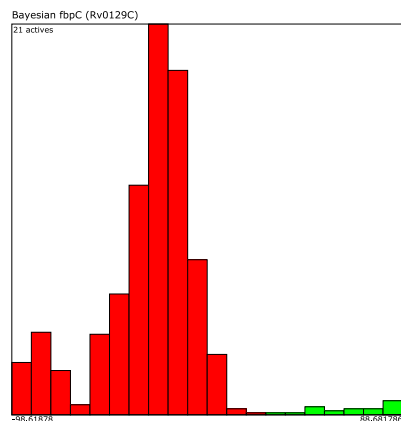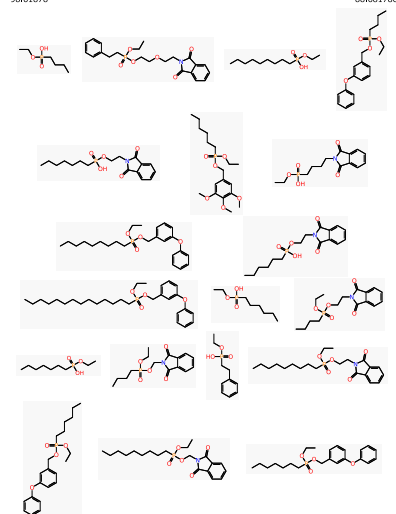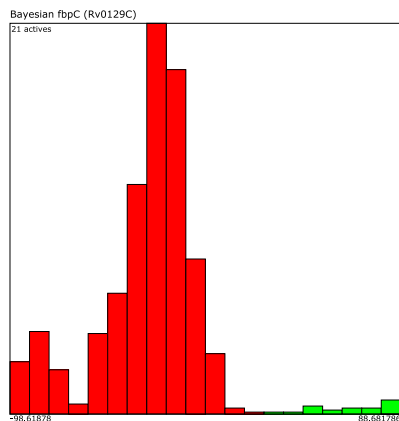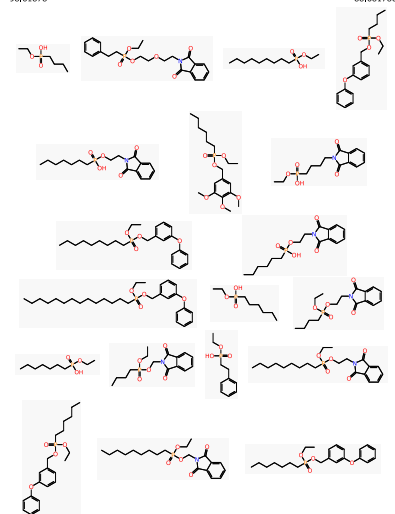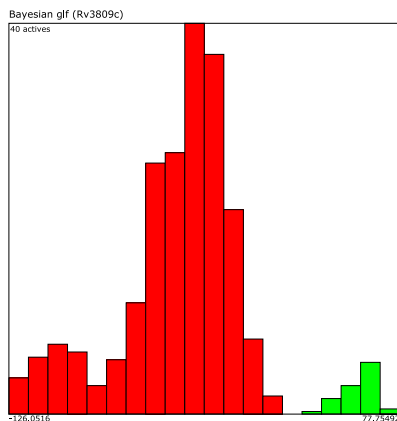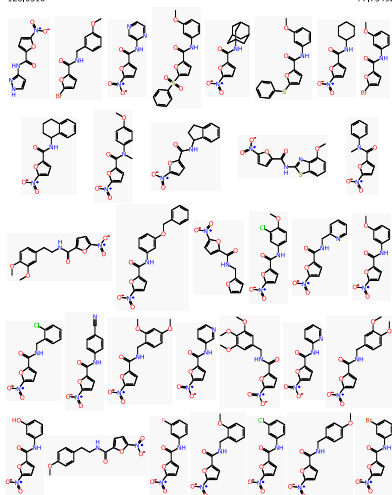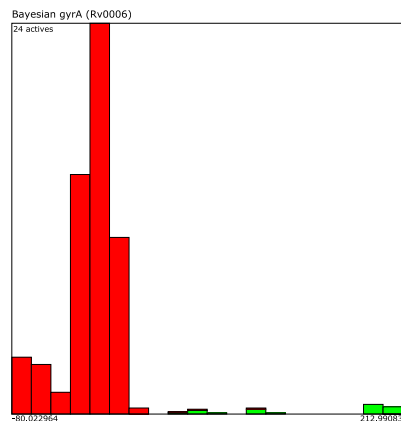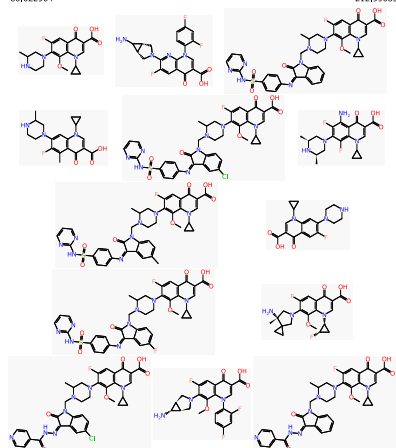

Supplement: Additional file 5: Table S4. — The Bayesian models for targets are shown in (a-e) and shows the target prediction charts and selected binders for targets with at least 3 examples. [file s13321-014-0038-2-S5.zip › 2034153920123298_add4a.pdf]

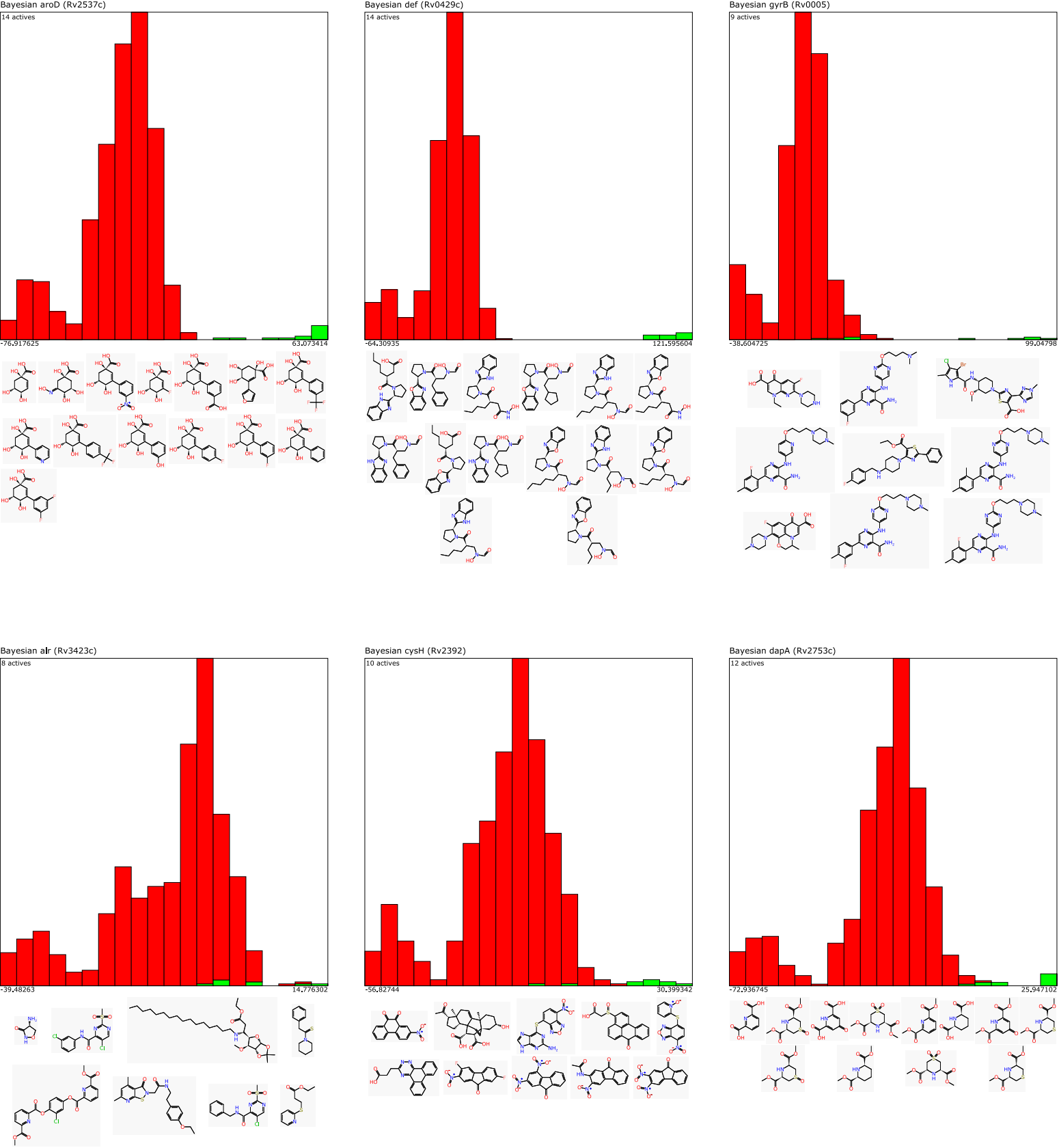

Supplement: Additional file 5: Table S4. — The Bayesian models for targets are shown in (a-e) and shows the target prediction charts and selected binders for targets with at least 3 examples. [file s13321-014-0038-2-S5.zip › 2034153920123298_add4c.pdf]

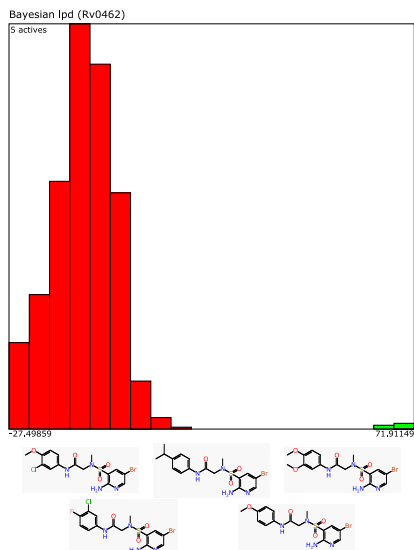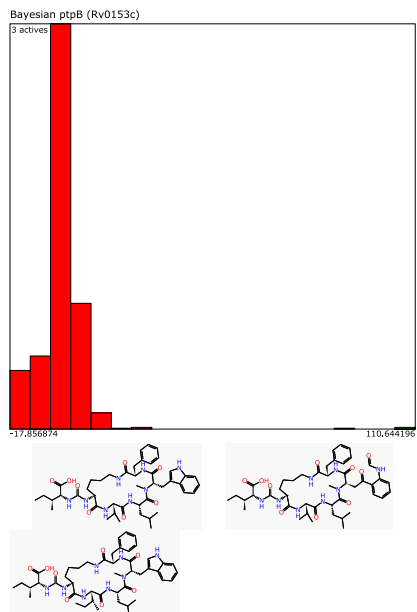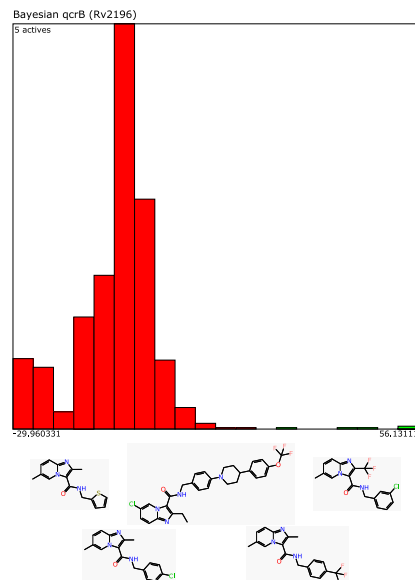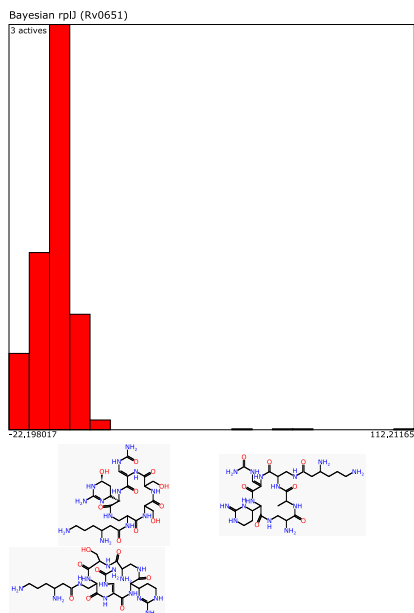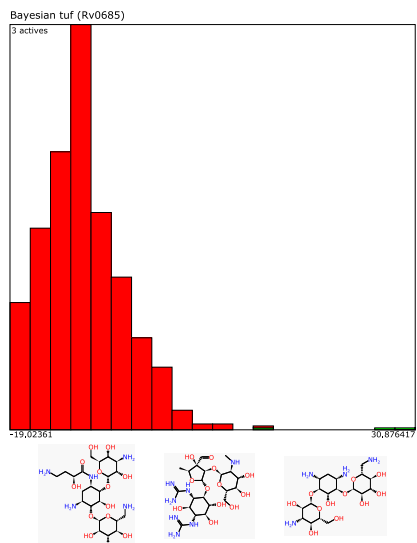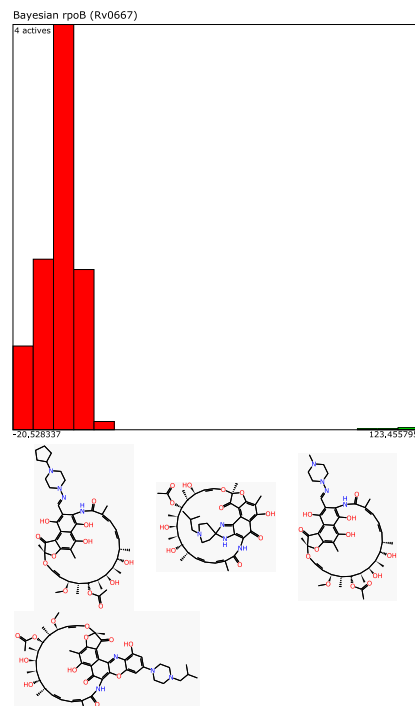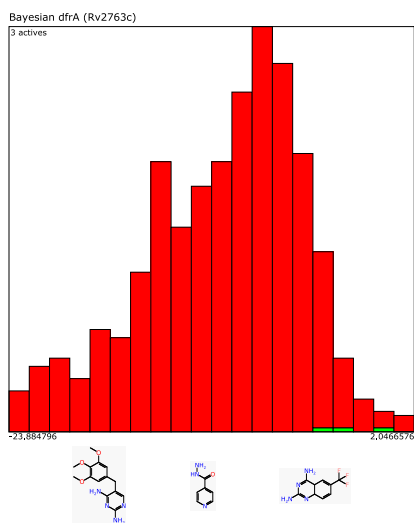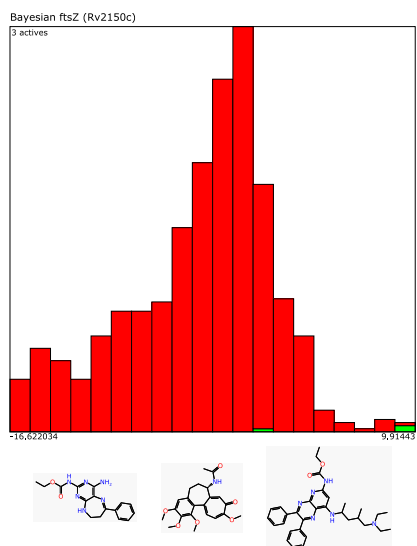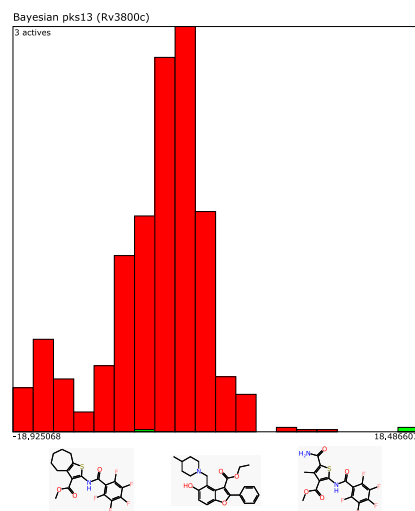

Supplement: Additional file 5: Table S4. — The Bayesian models for targets are shown in (a-e) and shows the target prediction charts and selected binders for targets with at least 3 examples. [file s13321-014-0038-2-S5.zip › 2034153920123298_add4e.pdf]
